# The impact of circulating preeclampsia-associated extracellular vesicles on the migratory activity and phenotype of THP-1 monocytic cells

**DOI:** 10.1038/s41598-018-23706-7

**Published:** 2018-04-03

**Authors:** Árpád Ferenc Kovács, Orsolya Láng, Lilla Turiák, András Ács, László Kőhidai, Nóra Fekete, Bálint Alasztics, Tamás Mészáros, Edit Irén Buzás, János Rigó, Éva Pállinger

**Affiliations:** 1 Department of Genetics, Cell- and Immunobiology, Semmelweis University, Budapest, Hungary; 2 MS Proteomics Research Group, Research Centre for Natural Sciences, Hungarian Academy of Sciences, Budapest, Hungary; 3 1st Department of Obstetrics and Gynaecology, Semmelweis University, Budapest, Hungary; 4 Seroscience Ltd, Budapest, Hungary; 5 Nanomedicine Research and Education Center, Institute of Pathophysiology, Semmelweis University, Budapest, Hungary; 6 MTA-SE Immunoproteogenomics Extracellular Vesicle Research Group, Budapest, Hungary

## Abstract

Intercellular communication via extracellular vesicles (EVs) and their target cells, especially immune cells, results in functional and phenotype changes that consequently may play a significant role in various physiological states and the pathogenesis of immune-mediated disorders. Monocytes are the most prominent environment-sensing immune cells in circulation, skilled to shape their microenvironments via cytokine secretion and further differentiation. Both the circulating monocyte subset distribution and the blood plasma EV pattern are characteristic for preeclampsia, a pregnancy induced immune-mediated hypertensive disorder. We hypothesized that preeclampsia-associated EVs (PE-EVs) induced functional and phenotypic alterations of monocytes. First, we proved EV binding and uptake by THP-1 cells. Cellular origin and protein cargo of circulating PE-EVs were characterized by flow cytometry and mass spectrometry. An altered phagocytosis-associated molecular pattern was found on 12.5 K fraction of PE-EVs: an elevated CD47 “don’t eat me” signal (p < 0.01) and decreased exofacial phosphatidylserine “eat-me” signal (p < 0.001) were found along with decreased uptake of these PE-EVs (p < 0.05). The 12.5 K fraction of PE-EVs induced significantly lower chemotaxis (p < 0.01) and cell motility but accelerated cell adhesion of THP-1 cells (p < 0.05). The 12.5 K fraction of PE-EVs induced altered monocyte functions suggest that circulating EVs may have a role in the pathogenesis of preeclampsia.

## Introduction

Extracellular vesicles (EVs) are nanosized particles enclosed by a phospholipid bilayer membrane. EVs been shown to mediate intercellular communication. EVs are significant players of autocrine, paracrine as well as endocrine signalling^1^. EVs are produced by living cells and can be detected in all biological fluids tested so far. In blood plasma, EVs are present in subpicomolar concentrations, with a size range within 100–1000 nm and around one gigadalton typical mass^2^. Based on their biogenesis and size distribution, EVs have been traditionally classified into at least three main subtypes: exosomes, microvesicles and apoptotic bodies^3^. In this manuscript we will use the term “12.5 K EVs” as a synonym of “microvesicles” – EVs enriched in 12.5 K pellets. Where it is relevant, the term “100 K EVs” is used to describe EV preparations enriched in “exosomes” – 100 K pellets as suggested by Théry *et al*.^4^.

The interaction of EVs with their potential target cells has been explored in several studies^5–7^, and their specific interaction with various immune cells has been also well documented^8–11^. Compared to the healthy, non-pregnant state, the circulating EV pattern is changed during pregnancy, and it is further modified in preeclamptic pregnancies^12,13^. Compared to the non-pregnant state a major increase in EV count can be observed in healthy pregnancies. Although the source of the EVs is heterogeneous^14^, one of the main source during pregnancy is the syncytiotrophoblast layer. The altered syncytiotrophoblast function in preeclampsia is also reflected by an even higher EV release^13^. The systemic inflammation induces a shift of EVs towards the sustainability of the pro-inflammatory and pro-coagulant activity characterized state^15^. Plasma EV pattern in preeclampsia is reflected by a significant increase in trophoblast derived EVs, monocyte derived EVs and increased tissue factor positive EVs compared to healthy pregnancy. A decrease in platelet derived EVs in preeclampsia has been systematically observed^16^.

Trophoblast-derived EVs originate from the feto-maternal interface and are present in the circulating EV pool. They can be detected by several methods, including flow cytometry (FC), confocal microscopy, mass spectrometry and transmission electron microscopy^11,17–19^.

However, the interaction between circulating EVs and immune cells during pregnancy and the subsequent functional consequences at the cellular level have not been fully explored yet. A few studies have addressed the role of placental EVs (*ex vivo* from placental explants, as well from trophoblastic cell line derived EVs) on immune cells^20–23^, showing an activation of immune cells, including blood monocytes^20,22^. EVs shed from preeclamptic placentas seems to be more pro-inflammatory^22,24^. Circulating monocytes are one of the most prominent environment- monitoring and sensing immune cells characterized by high plasticity, tissue infiltration capacity and cytokine production^25^. They have remarkable multipotency, and can differentiate into either inflammatory or anti-inflammatory subsets based on the surrounding stimuli^26^. Therefore, they contribute to immune homeostasis and may play a critical role in the pathogenesis of preeclampsia. Preeclampsia is a pregnancy-specific, immune-mediated inflammatory hypertensive disorder, characterized by altered circulating monocyte subsets^27^. Circulating monocyte subset distribution in preeclampsia is altered in comparison to healthy pregnancy: an increased number of intermediate monocytes (CD14hi/CD16+/HLA-DR+) together with a decreased classical monocyte subset (CD14hi/CD16-/CCR2hi) and increased non-classical monocyte subset(CD14low/CD16+/CCR2-/CCR5+)^28^. Normal pregnancy is characterized by a controlled systemic inflammatory reaction with progressive monocyte activation. This reaction is exaggerated in preeclampsia. However, the causes of the detected inflammatory reaction in both healthy and preeclamptic pregnancies are still unknown^20^.

In the present study we demonstrated that monocytes are target cells of pregnancy-associated EVs. So, we hypothesized that preeclampsia-associated EVs (PE-EVs) modify the function of THP-1 monocytic cell line and may have a role in the pathogenesis of preeclampsia. Results indicate that PE-EVs downregulate the migratory activity of THP-1 cells and induce an inflammatory phenotype of THP-1 cells. Our data also show that EVs isolated from blood plasma of preeclamptic patients are characterized by a modified exofacial protein pattern of EVs, a unique protein cargo and a dampened chemoattractant property. The data presented here highlight the impact of circulating blood plasma EVs on monocyte phenotype and function.

## Results

### Circulating EV pattern in healthy and preeclamptic pregnancy

To characterize the circulating EV pattern, first we assessed the size distribution of EV preparations. There were no significant differences between the healthy and preeclamptic plasma samples, as evaluated by dynamic light scattering (Suppl. Fig. 1A,B) and high-resolution flow cytometry (Suppl. Fig. 1C,D). EVs were further characterized by confocal laser scanning microscopy (CLSM) and conventional FC. The gating strategy for FC analysis is shown in Suppl. Figure 2. Isolated 12.5 K fraction of EVs were stained with fluorescent reporter molecule PKH26 (Suppl. Fig. 3A–B) and their vesicular nature was further confirmed by their sensitivity to 0.1% Triton X-100 detergent (Fig. S3C–D). Next we labelled the PKH26-stained EVs with Annexin V FITC and a trophoblast-specific (anti-human leukocyte antigen G – (HLA-G) APC conjugated)^29^ monoclonal antibody. Circulating EVs were also positive for CD63 (mean ± SEM: HP-EV = 3461 ± 715 PE-EV = 12778 ± 4698 p = 0.067 (n = 13) Suppl. Fig. S4A) vesicular marker protein, as evaluated by FC and their expression showed no difference between the healthy and preeclamptic samples. Other vesicular markers: Flotillin 2 (FLOT2) and Vesicular transport-associated clathrin (CLTC), as well as pregnancy-specific proteins: HLA-G, human leukocyte antigen E (HLA-E) and Pregnancy zone protein (PZP) were identified by mass spectrometry (Online Table 1). Immunophenotyping of circulating EVs revealed that most detected EVs were derived from platelets and their number was decreased in preeclamptic samples (26 622 ± 4 173 EVs/μL) compared to healthy pregnant- derived EVs (HP-EVs) (60 184 ± 5 504 EVs/μL). However, there was no difference between the amount of trophoblast-derived preeclamptic and healthy EVs (PE-EVs = 25 822 ± 2 030 EVs/μL; HP-EVs = 29 348 ± 2 380 EVs/μL, P > 0.05 (n = 15) Suppl. Fig. 4B). The relative distribution of the most abundant EV subpopulations detected in the EV pool were the phosphatidylserine positive EVs (HP-EV:31.5% PE-EV:16.9%), the platelet-derived EVs (HP-EV:40.2% PE-EV:27.4%), the trophoblast-derived EVs (HP-EV:19.6% PE-EV:26.5%) and tissue factor positive EVs (HP-EV:3.5% PE-EV:21.2%) (Suppl. Fig. 4C).

### Altered phagocytosis of preeclampsia-associated EVs is dependent upon the exofacial molecular pattern of EVs

**Figure 1:**
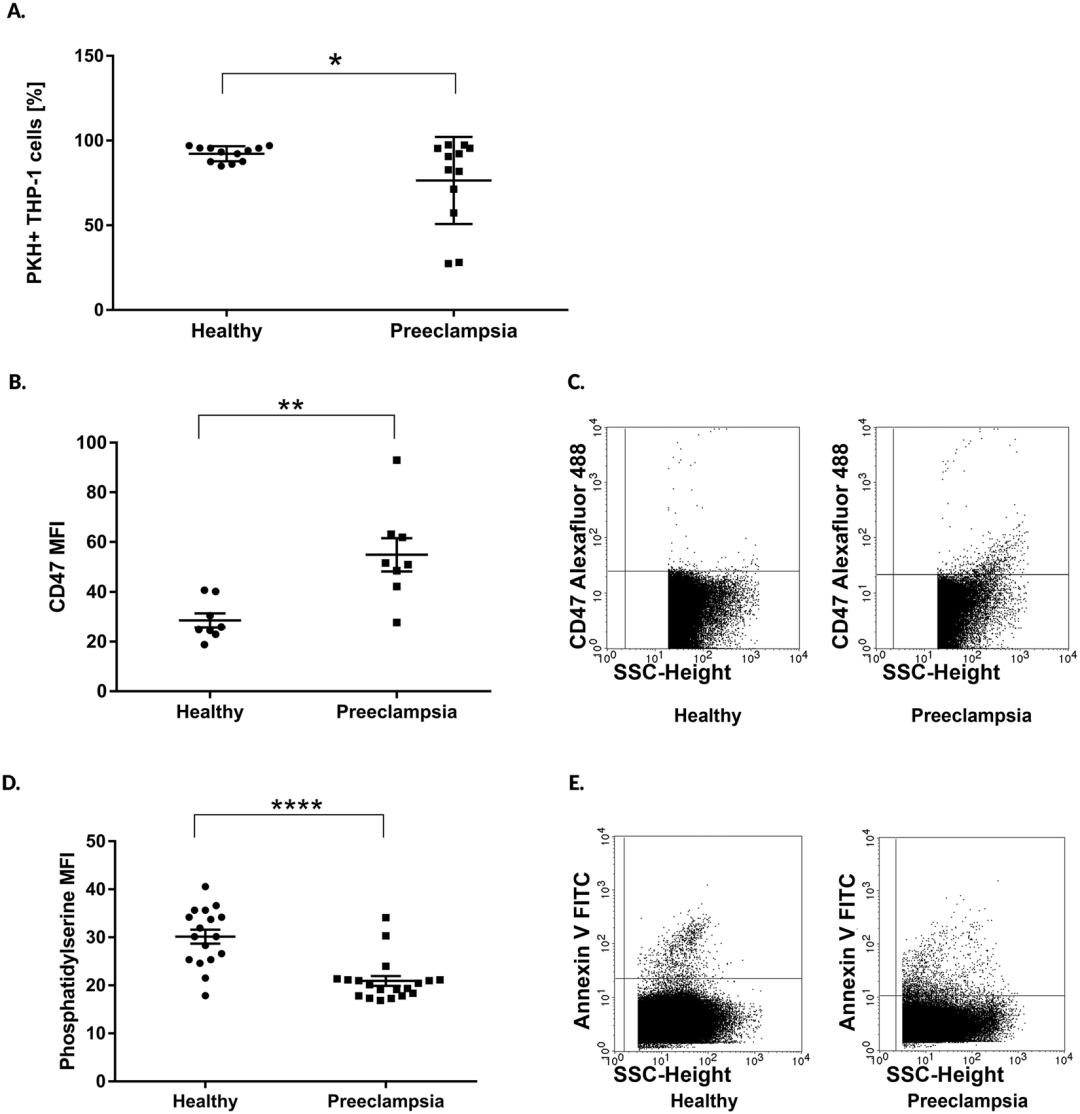
Phagocytosis of circulating extracellular vesicles by THP-1 monocytic cells. (**A**) Blood plasma 12.5 K fraction of EVs were labelled with PKH dye and their phagocytosis by THP-1 cells was quantified by flow cytometry. (**B)** Exofacial expression level of CD47 on EVs. (**C**) Representative dot plots of CD47 expression (**D)** Expression level of phosphatidylserine on circulating EVs. (**E**) Representative dot plots of Annexin V positive vesicles. The statistical analysis is based on two-sided Student’s *t*-test. ***p < 0.001, **p < 0.01, *p < 0.05. All data presented is expressed as mean ± SEM.

To study the interaction between EVs and THP-1 cells, we labelled the isolated 12.5 K fraction of EVs with the PKH26 dye and assessed EV binding to cells as well as EV phagocytosis by flow cytometry. PE-EVs and HP-EVs bound to THP-1 cells equally (PE-EVs = 13.95 ± 7.86%; HP-EVs = 5.24 ± 0.87%, P > 0.05). However, the elimination of PE-EVs by phagocytosis was significantly lower (percentage of phagocytosing THP-1 cells: PE-EVs = 76.44% ± 7.41; HP-EVs = 92.2% ± 1.28; P < 0.05; n = 12 patients/group Fig. 1A). We also performed the EV binding assay with primary monocytes isolated from healthy, non-pregnant women to confirm the *in vitro* data (Suppl. Fig. 5). To explore the background of the above results, we characterized the exofacial phagocytosis-associated molecules including CD47 as a “don’t eat me” signal and phosphatidylserine (PS) as an “eat-me” signal^30,31^. CD47 expression of PE-EVs was significantly higher compared to vesicles of healthy pregnant women (PE-EVs = 54.87 MFI ± 6.73; HP-EVs = 28.56 MFI ± 2.83; P < 0.005; Fig. 1B,C). Both the absolute number of circulating PS positive PE-EVs (PE-EVs = 15 783 events/μL ± 4 276; HP-EVs = 38 394 events/μL ± 9 922; P < 0.05) and the mean fluorescent intensity of Annexin V positive PE-EVs (PE-EVs = 20.93 MFI ± 1.0; HP-EVs = 30.14 MFI ± 1.4; P < 0.0001; Fig. 1D,E) were significantly lower in preeclamptic patients.

### PE-EVs induce decreased chemotactic and migratory activity and an increased adhesiveness of THP-1 cells

**Figure 2:**
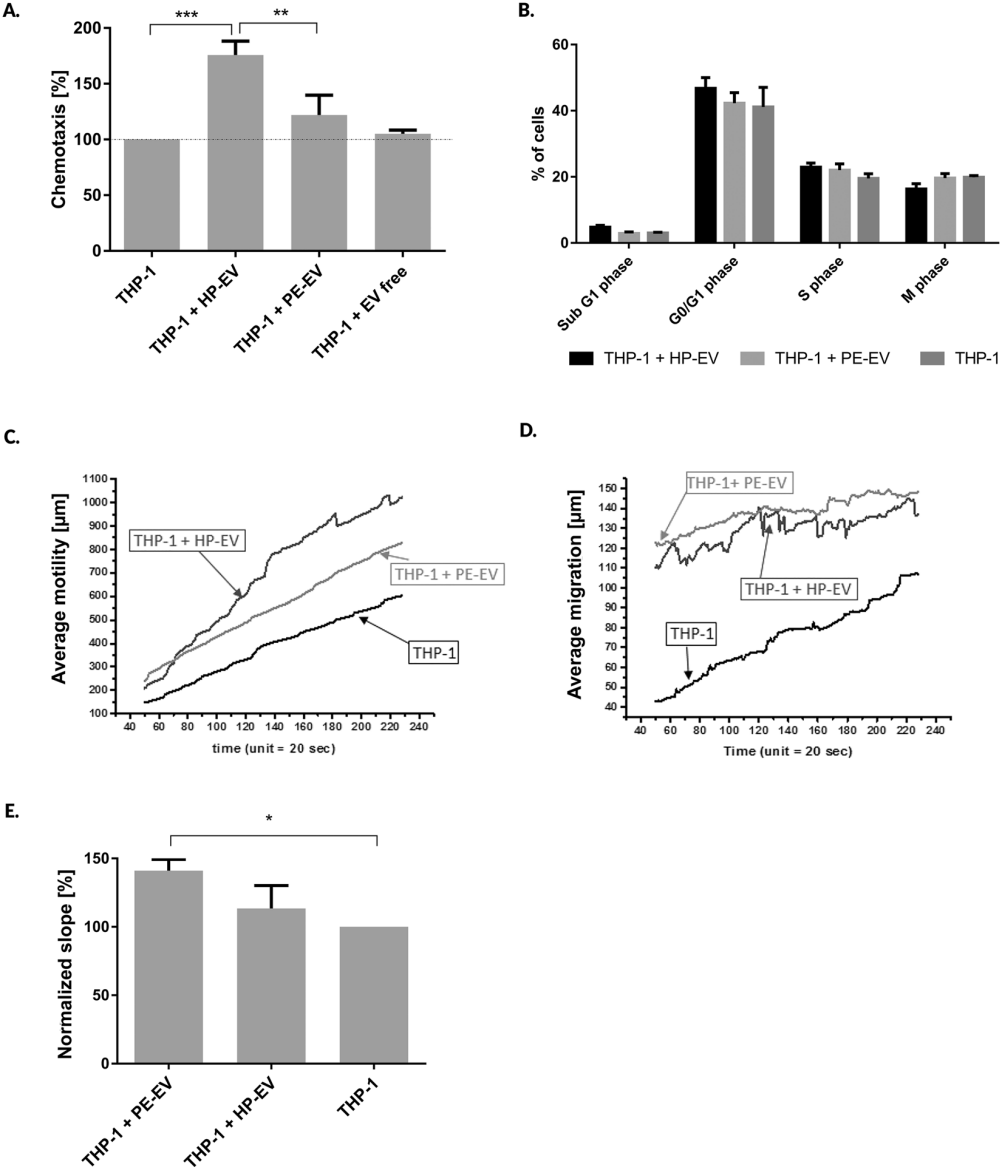
Assessment of the effects of pregnancy-associated EVs on the migratory and chemotactic activity and the adhesiveness of THP-1 cells. (**A**) The 12.5 K fraction of HP-EVs had significantly higher chemoattractant effect on THP-1 cells, as determined by Neuro Probe chemotaxis assay. (**B)** Flow cytometric cell cycle analysis shows that neither PE-EVs, nor HP-EVs induced cell proliferation in THP-1 cells. (**C)** Holographic microscopy analysis yielded a superior HP-EV (12.5 K fraction) induced THP-1 cell motility; (**D**) respectively both HP-EVs and PE-EVs (12.5 K fraction) influenced in a similar manner the average migration of THP-1 cells. (**E**) The 12.5 K fraction of PE-EVs induce a faster early adhesion of THP-1 cells. The statistical analysis is based on ANOVA test, followed by Bonferroni correction. ***p < 0.001, **p < 0.01, *p < 0.05. All data presented is expressed as mean ± SEM.

The chemotactic effects of circulating EVs were determined by a Neuro Probe chamber assay. As shown in Fig. 2A, the 12.5 K fraction of HP-EVs exerted a significant chemoattractant effect on THP-1 cells. Interestingly, the 12.5 K fraction of PE-EVs induced significantly lower chemotaxis (PE-EVs = 121.9% ± 17.86; HP-EVs = 175.8% ± 12.46; P < 0.05, Fig. 2A) in a concentration dependent manner (Suppl. Fig. 6). Neither 100 K fraction of EVs (PE group = 95.71% ± 10.10; HP group = 104.7% ± 7.49 P > 0.05) nor EV-depleted preparations (105.1% ± 3.3% P > 0.05) had a significant effect on THP-1 chemotaxis (Suppl. Fig. 6B). EV-induced cell proliferation was observed by a flow cytometric cell cycle analysis (to exclude unspecific results). The cell cycle analysis confirmed that neither PE-EVs nor HP-EVs induced cell proliferation (Fig. 2B).

For the evaluation of EV-induced (12.5 K fraction) migratory activity of THP-1 cells a holographic microscopy approach was used. THP-1 cell migration and motility were documented in real-time to observe the effects of 12.5 K fraction of PE-EVs. Significant motility and migration of THP-1 cells were induced by 12.5 K fraction of EVs (Fig. 2C,D). Of note, we found a lower cell motility after treatment with EVs isolated from preeclamptic plasma samples (PE-EVs = 3.22 ± 0.008; HP-EVs = 4.82 ± 0.06 slope, P < 0.05, Fig. 2C). In contrast, the average migration did not differ (PE-EVs = 0.142 ± 0.003; HP-EVs = 0.139 ± 0.006 slope, P > 0.05; Fig. 2D). We also determined the effects of pregnancy-associated EVs on the adhesiveness of THP-1 cells by a real-time impedance-based method. As an immediate effect, the 12.5 K fraction of PE-EVs induced a significantly more rapid cell adhesion (increased early adhesion) as compared to HP-EVs and the untreated cells. This rapid cell adhesion was characterized by an increased normalized slope. That is the corrected values of slope obtained from untreated cells (PE-EVs = 141.1% ± 8.26; HP-EVs = 113.4% ± 16.73; P < 0.05; Fig. 2E). Even after 24 hours of pre-incubation of THP-1 cells with 12.5 K fraction of EVs, a significantly higher adhesion could be detected in case of PE-EVs treated cells (normalized slope PE-EVs = 118.2% ± 2.19; HP-EVs = 94.21% ± 6.68; P < 0.05, not shown). The modified cell migratory pattern suggests an EV-induced THP-1 cell activation.

### PE-EVs induce tumour necrosis factor (TNF) production by THP-1 cells

**Figure 3:**
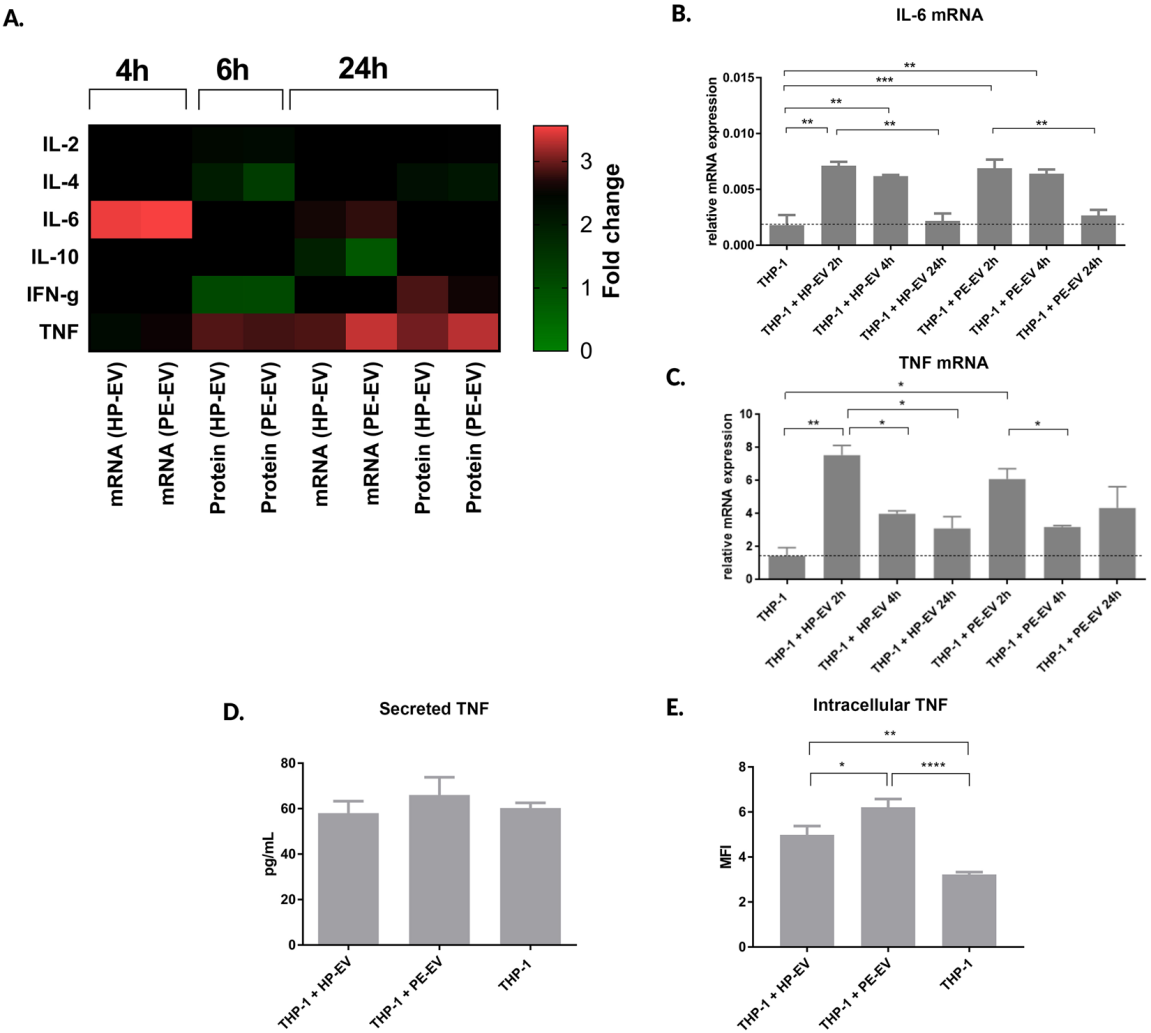
Cytokine mRNA and protein levels upon EV treatment. (**A**) Heatmap of THP-1 cells cytokine expression at mRNA and protein levels after 4, 6 and 24 hours of 12.5 K fraction of EV incubation with THP-1 cells. (**B)** IL-6 mRNA expression upon 12.5 K fraction of EVs treatment. (**C)** Secreted TNF expression levels in EVs treated cells as determined by a cytometric bead assay. (**D**) Intracellular TNF content was quantified after 24 hours of 12.5 K fraction of EVs incubation by flow cytometry measurement. Data is expressed in mean fluorescence intensity (MFI). Mann-Whitney U test; ***p < 0.001, **p < 0.01, *p < 0.05. Bars illustrate mean ± SEM.

The PE-EVs induced modifications of cytokine production by THP-1 cells were detected both at mRNA and protein levels (Fig. 3A). The IL-10 mRNA expression of 12.5 K fraction of PE-EV-treated THP-1 cells shows a slightly decreased fold change (0.89 ± 0.003). This effect is like those observed in the case of 12.5 K fraction of HP-EV-treated THP-1 cells (0.91 ± 0.05) and in untreated controls. In the case of IL-4 and IFN gamma, the mRNA expression was below the detection limit. However, the secreted cytokine levels were detectable (IL-4: PE-EVs = 50.43 ± 5.85 pg/mL; HP-EVs = 53.79 ± 6.44 pg/mL; IFN gamma: PE-EVs = 80.47 ± 10.68 pg/mL; HP-EVs = 123.3 ± 6.78 pg/mL). The most pronounced changes were observed in the cases of IL-6 and TNF (Fig. 3B). Although, both HP-EVs and PE-EVs (12.5 K fraction) enhanced the TNF mRNA levels, the effects of PE-EVs were higher (PE-EVs = 3.03 ± 0.89; HP-EVs = 2.16 ± 0.55 fold change). The changes of TNF production at protein level were also confirmed (Fig. 3D,E). The 12.5 K fraction of PE-EVs treatment induced elevation in the secreted TNF concentration (PE-EVs = 66.07 ± 7.75 pg / mL; HP-EVs 58.03 ± 5.3 pg / mL; P = 0.05) and significant intracellular TNF level increase (PE-EVs = 6.21 ± 0.37 MFI; HP-EVs = 4.98 ± 0.42 MFI; P < 0.05) compared to HP-EVs treatment. The 100 K fraction of EVs did not increase neither TNF, nor IL-6 gene expression (Suppl. Fig. 7).

### Unique migration and adhesion-associated protein cargo characterizes PE-EVs

**Figure 4:**
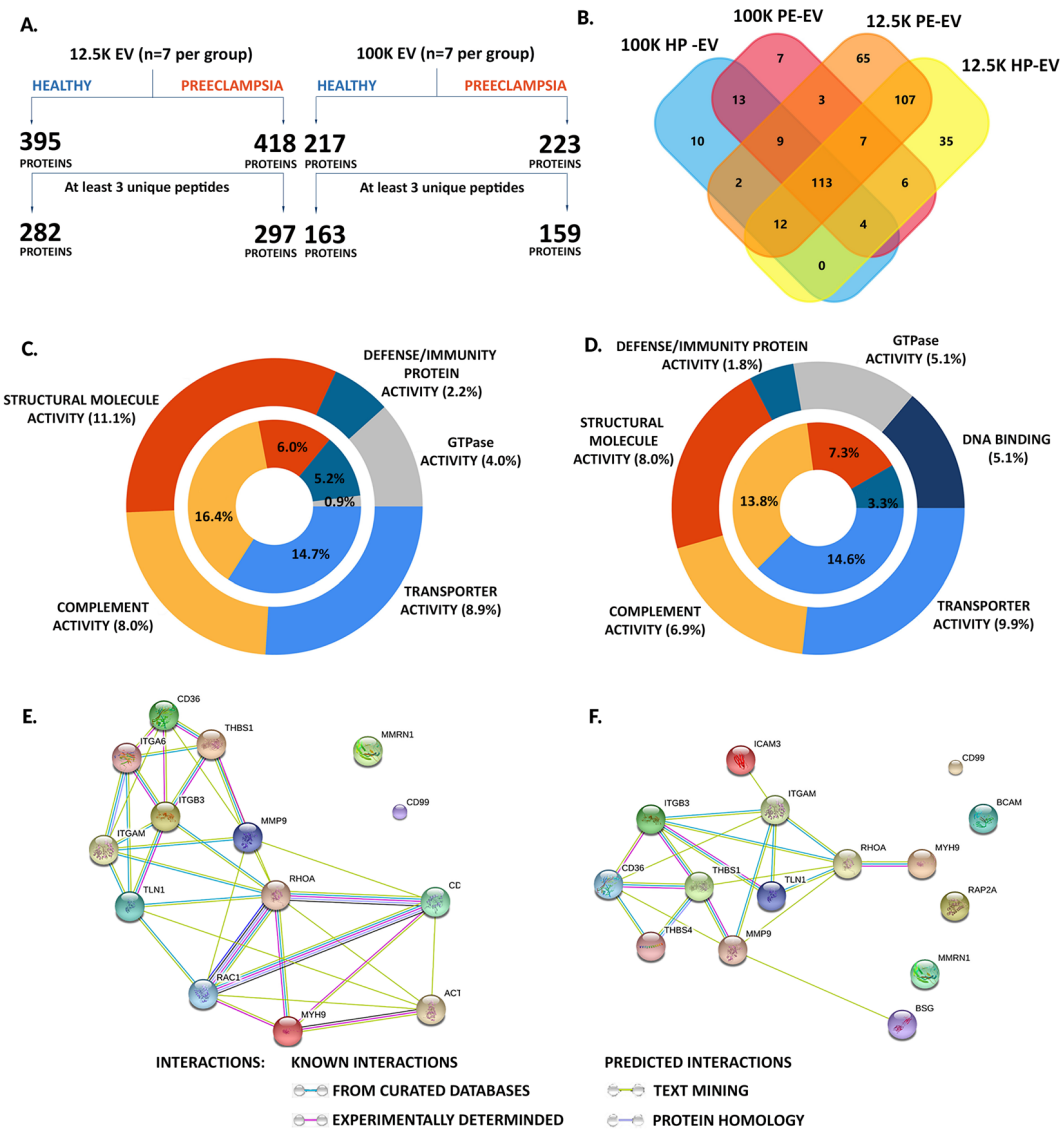
EV’s mass spectrometry – overview. (**A)** A total of 395 and 418 proteins were determined within the 12.5 K EVs pellet isolated from healthy or preeclamptic pregnants, respectively. The 100 K fraction of HP-EVs contained 217 proteins while 223 proteins could be detected in the 100 K EV pellet isolated from preeclamptic plasma. (**B)** Protein overlap between the studied groups. We identified common proteins in the EVs fractions: vesicular transport-associated clathrin (CLTC), rab proteins (RAB1a, RAB1b), flotillin 2 (FLOT2), adenylate cyclase-associated protein 1 (CAP1), and pregnancy related HLA-G, HLA-C, pregnancy zone protein (PZP), and pregnancy specific beta-1-glycoproteins (PSG) including: PSG6, PSG4, PSG11 could be detected in both 12.5 K fraction of HP-EVs and PE-EVs fractions. (**C)** The most prominent molecular functions for 12.5 K HP-EVs (on outer chart) and 100 K HP-EVs (on inner chart) pellets. (**D)** The most prominent molecular functions for 12.5 K PE-EVs (on outer chart) and 100 K PE-EVs (on inner chart) pellets. Predicted structural molecular activity is decreased in 12.5 K fraction of PE-EVs, compared to HP-EVs, meanwhile exclusively to PE-EVs fraction there are proteins with DNA binding capacity. The GTPase activity is significantly increased in both 12.5 K fraction of HP-EVs and PE-EVs compared to the 100 K fraction of HP-EVs and PE-EVs. (**E)** Migration and adhesion related protein interactome shows the protein interaction map between proteins involved in cell adhesion and migration identified in 12.5 K fraction of HP-EVs and (**F**) PE-EVs fractions, respectively. (STRING Database; http://string-db.org).

MS was performed to identify the causative proteins in isolated EV-fractions and was analysed according to the steps shown in Fig. 4A. To confirm and complete the analysis, we used the Panther (Protein ANalysis THrough Evolutionary Relationships)^32^ protein classification analysis and the String-database (http://string-db.org/) platforms. Proteins with the GO terms “extracellular vesicles” and “extracellular exosome” were assigned with the highest frequencies: 77%, 76%, 82% and 83% in 12.5 K fraction of HP-EVs, 12.5 K fraction of PE-EVs, 100 K fraction of HP-EVs and 100 K fraction of PE-EVs, respectively. FunRich enrichment analysis of the differentially expressed proteins associated with biological processes did not reveal any difference between preeclampsia and healthy pregnancy (Suppl. Fig. 8). On the other hand, both the composition and distribution of proteins related to cell migration and adhesion were characteristic for the tested groups (Suppl. Table 1). As shown in Fig. 4C,D, fewer proteins involved in structural molecular activity (PE-EVs: 8.0%; HP-EVs: 11.1%) or complement activity (PE-EVs: 6.9%; HP-EVs: 8.0%) were identified in PE-EVs compared to the control group. DNA binding proteins could be detected only in the preeclamptic samples (PE-EVs: 5.1%; HP-EVs: 0%). The adhesive glycoprotein thrombospondin-4 was unique in the PE-EVs fraction. The immunoregulatory Alpha-1-acid glycoprotein 2 and Protein S100-A9 proteins were more abundant in preeclamptic samples. The interaction map of proteins found in the 12.5 K fraction of HP-EVs (Fig. 4E) shows a heavily interconnected protein network that could explain the high migratory activity of THP-1 cells and the chemoattractant effect of these vesicles. The interaction map of migration and adhesion associated proteins of 12.5 K fraction of PE-EVs (Fig. 4F) reveals a reduced number of migration-associated proteins in this fraction as well as significantly weaker interprotein connections compared to HP-EVs. Additionally, the interactions between adhesion-related molecules were enhanced in correlation with the higher adhesion observed upon 12.5 K fraction of PE-EVs treatment.

## Discussion

Despite intensive efforts in the field of extracellular vesicles to better understand pregnancy complications, no EV-associated pathological pregnancy biomarker has been identified yet. On the other hand, only limited data are available about the effects of PE-EVs on circulating leukocytes such as monocytes. Advances in understanding of preeclamptic EVs-driven monocyte function may provide targets for therapeutic intervention and result in identification of new biomarkers for disease prediction.

Although many studies investigate the circulating EV pool during pregnancy^13,23,33^, there are no conclusive data regarding the absolute EV numbers or the cellular origin of EVs in pregnancy. In this study we detected a decreased total circulating EVs number and significantly lower amounts of platelet-derived EVs in preeclampsia compared to healthy pregnant women. These data correlate with the well-known reduction of platelet number observed in preeclamptic patients^34^. Furthermore, it may also refer to an accelerated removal via binding to circulating white blood cells such as T lymphocytes^9^.

The interaction between EVs and monocytes and its subsequent functional consequences were mostly studied in the cases of tumour derived EVs^35^. Furthermore, an enhanced mesenchymal stem cell recruitment by dendritic cells derived EVs was reported^36^. In the context of reproductive immunology, it has been described that (1) placenta-derived EVs in co-culture with non-pregnant peripheral blood mononuclear cells (PBMCs) bound to monocytes and B cells^37^, (2) trophoblast-derived EVs recruited monocytes and influence their function at the feto-maternal interface^38^, (3) plasma-derived 100 K fraction of EVs from healthy pregnant women induced increased migration of endothelial cells *in vitro*^18^, (4) EVs derived from the Swan71 trophoblastic cell line increased monocyte migration^21^ and (5) circulating 100 K fraction of EVs from healthy pregnants increased cell migration *in vitro*. Despite the available data about EV-monocyte interaction or EV-induced monocyte functional changes, these interactions between monocytes and circulating EVs from preeclamptic pregnancies have not been studied yet.

This current study represents the initial demonstration that the 12.5 K fraction of PE-EVs interact with monocytes and modify their functionality, including migratory activity and cytokine production. Comparison of the effects of 12.5 K fraction of PE-EVs with HP-EVs demonstrates that both the chemotactic activity and the motility of THP-1 cells were significantly suppressed after PE-EVs induction, and their adhesion was significantly more rapid.

By matching the identified 12.5 K fraction of HP-EVs protein cargo with Exocarta, EVpedia and Vesiclepedia databases^39–41^, we could detect 34 proteins not reported previously in EVs, and 56 unreported proteins in the same fraction isolated from the plasma of preeclamptic women. Importantly, we found several proteins reported to be involved in cell migration. The 12.5 K fraction of HP-EVs associated myosin-9 (MYH9) and ras-related C3 botulinum toxin substrate 1 proteins (RAC1) together with integrin alpha-6, integrin beta-3 and integrin alpha-M could promote and directly regulate cell migration in an integrin dependent manner and could modify the cell directionality through cell division control protein 42 homolog protein (CDC42) present in the vesicular cargo (Suppl. Table 1).

Tong and colleagues^42^ have analysed the proteomic cargo found in trophoblast derived EVs isolated from first trimester placentas. They have showed the presence of inflammatory proteins as well as the presence of the CD47 molecule (“don’t eat me signal”) in the EV fractions. They have also identified the CDC42, MYH9 and RAC1 proteins as part of the protein cargo. Their result suggests, that the identified key proteins that play an essential role in the altered EV induced detected cellular responses (based on our *in silico* analysis of mass spectrometry data) to be of placental EV origin. Furthermore, one group who focuses on preeclampsia research, discusses in a meta-review analysis the proteome of trophoblast derived EVs. They highlight the presence of inflammation and apoptosis related proteins in the vesicular cargo. However there is a little overlap between trophoblastic cell line (Swan71, HTR8/SVneo) derived and primary trophoblast derived EV proteome, which may indicate, that *in vivo* the EV cargo may present a very high plasticity^43^.

We showed that 12.5 K fraction of HP-EVs were superior to PE-EVs in inducing THP-1 cell migratory activity, as detected by cell migration, motility, chemotaxis and adhesion. Salomon *et al*. showed that plasma exosomes (100 K fraction of EVs) from healthy pregnant women induced increased migration of endothelial cells *in vitro* compared to 100 K fraction of EVs isolated from non-pregnant women^18^. However, in the above study the authors did not test preeclamptic samples. The results of our functional studies consistently support that isolated EVs from plasma samples are biologically active and exert their effects in a concentration dependent manner.

It has been previously described, that inflammatory responsiveness is increased in pregnancy, and EVs from placental origin have proinflammatory potential^23^. PBMCs treated with EVs derived from a trophoblastic cell line cultured under hypoxic conditions, produced increased levels of IL-6 and TNF^44^. It has been suggested that placental EVs induce monocyte activation, resulting in increased production of IL-8, IL-6 and IL-1 beta^20^. EVs isolated from maternal perfusion of placental cotyledon induced intracellular production of TNF, IL-12p70 and IL-18 in monocytes^23^. Our present work is, in general, consistent with data of Sokolov *et al*. The authors evaluated the effects of circulating EVs during healthy and preeclamptic pregnancies on the protein expression of THP-1 cells^45^. The results suggest that circulating EVs may contribute to THP-1 cell migration. According to this study HP-EVs induce an activated phenotype of THP-1 cells, characterized by decreased CD181 expression. The authors observed that PE-EVs influenced CD181 expression to a lesser degree. They also suggest that the impact of PE-EV treatment on THP-1 cell surface adhesion proteins could modify cell adhesion and chemotaxis, however, no further functional assays were performed. Of note, the two studies should be compared with cautiousness as Sokolov *et al*. isolated blood plasma EVs from heparin anticoagulated blood samples, while we collected blood samples in Anticoagulant Citrate Dextrose Solution (ACD-A) tubes in order to minimize the *in vitro* generation of EVs from platelets^46^.

All these data point towards that PE-EVs have the capacity to activate monocyte cells and this activation has significant functional consequences on the microenvironment. Another possibility is that EVs derived from activated monocytes will induce further effects on the target cells, leading to the complex hallmark of intercellular communication.

In conclusion, our present study suggests that circulating EVs activate THP-1 monocytes, induce intra-monocytic TNF expression, as well as an altered chemotaxis and cell migration pattern. This is the first study to investigate in preeclampsia the effects of circulating EVs on monocyte chemotaxis, cell adhesion and migration *in vitro*. *S*everal studies (including ours) demonstrate, that EVs, despite their heterogeneity, can play highly specific roles in an orchestrated manner.

Further studies could untangle new pathways of the described functional effects on monocytes by profiling the miRNA or other small RNA cargo of EVs by new generation sequencing. This could open new tracks of research on the horizontal nucleic acid transfer between the placenta and maternal immune cells in healthy and preeclamptic pregnancies. On one hand, analysis of EV-treated monocyte – endothelial cell interaction could decipher the potential vascular damage observed in preeclampsia. On the other hand, the modified migratory activity may be the cause of the altered macrophage distribution found in preeclamptic placentas. These studies could also contribute to a better understanding of the molecular mechanisms involved in the pathogenesis of preeclampsia.

## Methods

### Study groups and plasma sampling

Blood samples from both healthy and preeclamptic third trimester pregnant women were obtained at the 1st Department of Obstetrics and Gynaecology at Semmelweis University, Budapest, Hungary. Normotensive patients were age and gestational age matched with no detectable proteinuria or history of preeclampsia. Exclusion criteria were the presence of any chronic disease or acute infection in either group (Suppl. Table. 2). Blood samples were collected in acid-citrate dextrose tubes (ACD-A tube, Greiner Bio-One) to prevent *in vitro* generation of EVs in the blood collection tube^46^. Blood was centrifuged at 800 *g* for 5 minutes to obtain plasma. Platelet-poor plasma was separated by centrifugation at 2 500 *g* for 15 minutes at room temperature. Next, it was recentrifuged once again at 2 500 *g* for 15 minutes to obtain platelet-free plasma (PFP). The PFP aliquots were stored at −80 °C until use. Each experiment was performed in triplicates and with a minimum of 6 patient samples/group. During the entire investigation period, we followed the guidelines and regulations of the Helsinki Declaration in 1975, and the experiments were approved by the Hungarian Scientific and Research Ethics Committee; all tested individuals signed an informed consent form.

### Extracellular vesicle isolation

EVs were isolated by serial centrifugation and ultracentrifugation from 1 mL of PFP diluted with 1.5 mL phosphate-buffered saline (PBS) filtered on a 0.22 μm membrane (Millipore). The 12.5 K EV pellet was sedimented at 12 500 *g* for 20 minutes at 16 °C (Z216 M K Microlite centrifuge, fixed angle 200.88 rotor, Hermle Labortechnik GmbH, Wehingen, Germany) and subsequently washed at 12 500 *g* for 15 minutes at 16 °C. The 100 K EV pellet was sedimented from the first 12.5 K pellet supernatant at 100 000 *g* for 70 minutes at 4 °C (Optima MAX-XP, fixed angle MLA-55 rotor, Beckman Coulter Inc, Brea, USA). The obtained pellet was washed by filtered (0.22 µm pore size, Millipore) PBS at 100 000 *g* for 70 minutes at 4 °C (Suppl. Fig. 9). EV-depleted fraction was obtained from the supernatant of 100 K pellet by centrifugation at 100 000 *g* for 4 hours at 4 °C and filtered through a 0.22 μm filter. Following the recommendations of the position paper of International Society for Extracellular Vesicles (ISEV)^47^ both fractions were characterized qualitatively and quantitatively by several methods including by high-resolution flow cytometry (hrFC), dynamic light scattering (DLS), confocal laser scanning microscopy (CLSM). Annexin V and anti-CD63 binding were assessed by FC. In selected experiments (DLS, CLSM, Chemotaxis assay), EVs were also isolated with qEV size exclusion columns (IZON, Izon Science Ltd.) following the manufacturer’s instructions. Briefly, 500 µL of fractions 7, 8 and 9 were collected under sterile conditions, and the pooled fractions were analysed.

### Flow cytometry (FC) of EVs

Platelet free plasma samples 3 μL/sample was diluted in 300 μL 0.2 μm filtered sterile PBS and incubated with pre-titrated fluorochrome conjugated antibodies. After 15 minutes of incubation the samples were measured. As control unstained diluted plasma samples, mock control (same concentration of fluorochrome conjugated antibody without sample) were used. To confirm the vesicular nature 0.1% Triton X-100 (Sigma-Aldrich) detergent was also used to confirm the vesicular nature of events, as described by György *et al*.^48^. Only the events which disappeared upon detergent addition were considered EVs (Suppl. Fig. 3). Annexin V staining was carried out in the presence of 2.5 mM Ca^2+^.

Absolute counts of circulating EVs were calculated using Count Check Microbeads (Sysmex Partec GmbH, Germany). EV absolute counting with flow cytometer was done with the following formula: number of vesicles in sample = (detected EV events in the EV gate minus Triton X-100 resistant events/number of events in bead gate) × absolute count of Count Check beads in the tube × plasma dilution

Measurements were carried out using a BD FACSCalibur (BD Biosciences, San Jose, CA, USA) and an Apogee A50 Micro (Apogee Flow Systems Ltd) FC. The hrFC has an improved minimum detection size, reflected by detection of different submicron sized beads with a refractive index (R = 1.42) like EVs. Optimization of cytometer settings and gating strategy is detailed in Suppl. Fig. 2.

Isolated 12.5 K EV pellets were labelled with freshly prepared PKH26 membrane dye according to the manufacturer’s instructions (Sigma, St Louis, MO, USA). The isolated EV fraction was resuspended in 100 μL Diluent C. Next, 5 μM concentration of PKH26 dye was added, followed by 15 minutes of incubation at room temperature in the dark. The staining reaction was stopped by the addition of 100 μL 4% Bovine Serum Albumin and incubated for 1 minute to allow the binding of the excess dye (Protein solution is suitable to inhibit of unbound dye aggregation and to allow binding of excess dye). 1 mL of cell culture medium was added and the PKH26 labelled vesicles were washed by centrifugation at 12 500 g for 15 minutes at 16 °C. The effectiveness of staining was evaluated by flow cytometry (detection of PKH fluorescence signal inside EV gate) and CLSM. Measurement of diluted PKH dye solution without vesicles (mock control) and differential detergent lysis were applied for validation. The effectiveness of staining was evaluated by FC and CLSM. Measurement of diluted PKH dye solution without vesicles (mock control) and differential detergent lysis were applied for validation. (Suppl. Fig. 3).

### Mass spectrometry (MS)

EV pellets for MS analysis were resuspended in 25 μL HPLC water. Vesicle preparations were subjected to repeated freeze-thaw cycles and digested in solution as previously described^49^.The resulting peptides were cleaned using PierceTM C18 spin columns (Thermo Fisher Scientific, Waltham, MA, USA). Peptides were analysed using a Waters nanoAcquity UPLC or a Dionex Ultimate 3000 RSLCnano system coupled to a Bruker Maxis II Q-TOF mass spectrometer (Bruker, Bremen, Germany) with CaptiveSpray nanoBooster ionization source. Following trapping, peptides were separated on a 25 cm Waters Peptide BEH C18 nanoACQUITY 1.7 µm particle size UPLC column using gradient elution. Data processing was performed with ProteinScape 3.0 software (Bruker Daltonik GmbH, Bremen, Germany). Proteins were identified using Mascot (version Mascot 2.5; Matrix Science, London, UK) search engine against the Swissprot database (2015_08). The following parameters were used: Homo sapiens taxonomy, trypsin enzyme, 7 ppm peptide mass tolerance, 0.05 Da fragment mass tolerance, 2 missed cleavages. Carbamidomethylation was set as fixed modification, while deamidation (NQ) and oxidation (M) as variable modifications. To identify key networks, genome ontology (GO) term enrichment was performed on the identified proteins. For gene ontology (GO) and pathway analysis we used FunRich software (Uniprot and Funrich databases)^50^. The mass spectrometry proteomics data have been deposited to the ProteomeXchange Consortium via the PRIDE^51^ partner repository with the dataset identifier PXD008665.

### Cellular responses induced by EVs on THP-1 cells

THP-1 human monocytic cell line (ATCC® TIB-202™) was used in the study. THP-1 cells were cultured in FBS-free RPMI 1640 medium for 24 hours before functional experiments. Cell viability was determined before functional assay by propidium iodide (PI) staining^52^ (Suppl. Fig. 10).

To detect cell binding of EVs, blood plasma-derived PKH26 labelled EVs (1 × 10^6^) were added to THP-1 cells (5 × 10^5^) for 30 minutes at 4 °C for the investigation of EV’s binding. The binding of PKH26 labelled EVs to THP-1 cells was quantified as the PKH fluorescence intensity parameter (geometric mean values - MFI) inside the THP-1 gate (the mock control did not get any signal intensity at PKH wavelength). THP-1 cells were incubated with PKH- labelled EVs in the presence of 4% 0.2 μm filtered Bovine Serum Albumin containing PBS for 30 minutes at 4 °C. As negative control unstained samples and diluted PKH dye solution without vesicles (mock control) were applied.

The detection of phagocytosis was realized via the incubation of PKH26 labelled EVs (1 × 10^6^) with THP-1 cells (5 × 10^5^) for 30 minutes at 37 °C. Phagocytic capacity was evaluated by the mean fluorescence intensity of PKH26 within the THP-1 gate.

The effects of plasma-derived EVs on cell proliferation (1 × 10^6^ EVs/5 × 105 THP-1 cells) were detected by PI staining of DNA content of THP-1 cells^53^.

The effect of isolated EVs on cell adhesion (8 × 10^4^ EVs/4 × 10^4^ THP-1 cells/well) was assessed using the xCELLigence SP System (Roche Applied Science, Indianapolis, IN, USA). The 96 well E-plate was coated with 5 μg/mL human fibronectin (diluted in distilled water, Millipore). The impedimetric measurement was performed as previously reported^54^. Ten thousand cells per well were seeded. The adhesion of THP-1 cells was monitored every 20 seconds for 24 hours. Adhesion of the cells was described by the slope of Cell index (CI) calculated for a 3-hour time interval. The slope values were expressed in percentage of the control.

Chemotactic responsiveness of THP-1 cell line was measured in a two-chamber assay, Neuro Probe MBB 96 chamber (Neuro Probe, Gaithersburg, MD, USA) by using polycarbonate filters with 5 μm pore size. The EV pellets were diluted in FCS-free cell culture medium and were incubated for 3 hours at 37 °C in a humidified 5% CO_2_ atmosphere. The number of positive chemotactic responder cells was detected by AlamarBlue assay. Identical points of the concentration course study represent average of 8 parallel measurements. The registered value was normalized to the control and this value is given as “Chemotaxis index” (Chtx. ind.), in percent:

$$Chtx.ind[ \% ]=(({\overline{OD}}_{560,A}\mbox{--}{\overline{OD}}_{590,A})/({\overline{OD}}_{560,Ctrl}-{\overline{OD}}_{590,Ctrl}))\times 100.$$

Fresh culture medium and EV-free solution served as control substances in simultaneous runs.

The HoloMonitor M4 (Phase Holographic Imaging, Lund, Sweden) was used as an incubator-proof time-lapse and cell tracking system for adherent cells. In the present experiments the chemokinetic effects were evaluated by holographic tracking, measurement and calculation of the following parameters: (1) migration: the shortest direct distance from the starting point to the end point (μm); (2) motility: the actual way travelled from the starting point to the end point (μm). THP-1 cells (4 × 10^4^) were seeded on a Petri dish (35 mm diameter) and cultured for 24 h. After the incubation resting THP-1 cells were monitored for cell migration and motility for 2 hours (control THP-1 cells). Next, 12.5 K fraction of HP-EVs or PE-EVs were added to the resting cells and migration and motility activities of the cells were documented (HP-EVs or PE-EVs treated cells). Cell migration was normalized to the untreated resting THP-1 cells (Suppl. Fig. 11). In time-lapse tracking the settings were: total time = 2 h; interval: 30 sec. For evaluation of images of time-lapse automatic background thresholding method was used with “Minimum error sets” algorithm (adjustment = 128) using the minimum error histogram-based threshold method; in object definition, the minimum error object size was 36. The number of evaluated cells/image: 50; the total number of evaluated images: 240. For manual cell identification, objects of the marginal zone were abandoned. For holographic microscopy HoloStudio M4 2.5 program was used to analyse data.

Blood plasma-derived EVs (1 × 10^6^) were incubated with THP-1 cells (5 × 10^5^) for 2, 4, 6 or 24 hours for evaluation of EV-induced cytokine production. The effects of EVs on cytokine production were determined at mRNA and/or protein levels.

### mRNA transcript detection

For gene expression analysis, first total RNA was isolated from untreated and EV-treated THP-1 cells and was purified with a Total RNA Mini Kit (Geneaid Biotech Ltd., Taipei, Taiwan). One μg RNA was reverse transcribed with Sensifast cDNA Synthesis Kit (Bioline, London, UK). The RNA concentration and purity of each sample were determined a NanoDrop ND-1000 Spectrophotometer (NanoDrop Technologies, Inc., Wilmington, Delaware). Samples were stored at −80 °C until cDNA synthesis. Quantitative PCR reactions using SensiFAST Probe Hi-ROX Kit with Taqman probes were carried out on an ABI 7900 real-time PCR instrument according to the manufacturer’s instructions. Real-time PCR results were calculated according to the following protocol: relative expression level = 2^−∆Ct^, where ∆Ct = Ct (of gene of interest) − Ct (of housekeeping gene) with HPRT as endogenous control.

For the detection of EV-induced cytokine secretion by THP-1 cells we used two different methods: a membrane-based sandwich immunoassay (Human Cytokine Array Panel A Kit, R&D Systems; Minneapolis, MN, USA) and a flow cytometric bead-based immunoassay (Human Th1/Th2 Cytokine Kit; Becton Dickinson, USA). GMS array scanner software was applied for analysis of membrane-based assay. Positive control signals on each array were used for normalization, and baseline fold-change differences were calculated between untreated and EV-treated samples. TNF production of EV-induced THP-1 cells was confirmed by the intracellular TNF staining of cells.

### Statistical analysis

GraphPad Prism version 7.0 (GraphPad Software, La Jolla California, USA) was used for statistical analysis. For normally distributed data Two-sided Student’s unpaired *t*-test, respectively for multiple parameter analysis ANOVA test was used, followed by Bonferroni correction. For data not showing normal distribution Mann-Whitney U test was used. The level of significance was set at p < 0.05.

The datasets generated during and/or analysed during the current study are available from the corresponding author on reasonable request.

## Electronic supplementary material

Supplementary information

Mass spectrometry protein data
