# Author Correction: The impact of circulating preeclampsia-associated extracellular vesicles on the migratory activity and phenotype of THP-1 monocytic cells

**DOI:** 10.1038/s41598-018-29611-3

**Published:** 2018-07-31

**Authors:** Árpád Ferenc Kovács, Orsolya Láng, Lilla Turiák, András Ács, László Kőhidai, Nóra Fekete, Bálint Alasztics, Tamás Mészáros, Edit Irén Buzás, János Rigó, Éva Pállinger

**Affiliations:** 1 Department of Genetics, Cell- and Immunobiology, Semmelweis University, Budapest, Hungary; 2 MS Proteomics Research Group, Research Centre for Natural Sciences, Hungarian Academy of Sciences, Budapest, Hungary; 3 1st Department of Obstetrics and Gynaecology, Semmelweis University, Budapest, Hungary; 4 Seroscience Ltd, Budapest, Hungary; 5 Nanomedicine Research and Education Center, Institute of Pathophysiology, Semmelweis University, Budapest, Hungary; 6 MTA-SE Immunoproteogenomics Extracellular Vesicle Research Group, Budapest, Hungary

Correction to: *Scientific Reports* 10.1038/s41598-018-23706-7, published online 03 April 2018

The Acknowledgements section in this Article is incomplete.

“We are grateful for Ms. Andrea Orbán’s technical assistance. This work was funded by the Hungarian Scientific Research Fund (OTKA K113023, 11958, 120237 and PD121187), NVKP_16-1-2016-0017 and VEKOP-2.3.3-15-2017-00016). Árpád Ferenc Kovács is a Kerpel-Fronius Ödön Fellow. Lilla Turiák would like to acknowledge the Bolyai Janos Research Scholarship program.”

should read:

“We are grateful for Ms. Andrea Orbán’s technical assistance. This work was funded by the Hungarian Scientific Research Fund (OTKA K113023, 11958, 120237 and PD121187), NVKP_16-1-2016-0017, VEKOP-2.3.3-15-2017-00016 and EFOP-3.6.3-VEKOP-16-2017-00009). Árpád Ferenc Kovács is a Kerpel-Fronius Ödön Fellow. Lilla Turiák would like to acknowledge the Bolyai Janos Research Scholarship program.”

