# Supplementary material for: The impact of circulating preeclampsia-associated extracellular vesicles on the migratory activity and phenotype of THP-1 monocytic cells
Source: Sci Rep. 2018 Apr 3;8:5426. doi: 10.1038/s41598-018-23706-7 (PMC5882809; doi:10.1038/s41598-018-23706-7)
Supplement: Supplementary file 1 — Supplementary information [file 41598_2018_23706_MOESM1_ESM.docx]

Title: The impact of circulating preeclampsia-associated extracellular vesicles on the migratory activity and phenotype of THP-1 monocytic cells

Árpád Ferenc Kovács^1^, Orsolya Láng^1^, Lilla Turiák^2^, András Ács^2^, László Kőhidai^1^, Nóra Fekete^1^, Bálint Alasztics^3^, Tamás Mészáros^4,5^, Edit Irén Buzás^1, 6^, János Rigó Jr^3^, Éva Pállinger^1^

^1^ Department of Genetics, Cell- and Immunobiology, Semmelweis University, Budapest, Hungary

^2^ MS Proteomics Research Group, Research Centre for Natural Sciences, Hungarian Academy of Sciences

^3^ 1st Department of Obstetrics and Gynaecology, Semmelweis University, Budapest, Hungary

^4^ Seroscience Ltd, Budapest, Hungary

^5^ Nanomedicine Research and Education Center, Institute of Pathophysiology, Semmelweis University, Budapest, Hungary

^6^ MTA-SE Immunoproteogenomics Extracellular Vesicle Research Group, Budapest, Hungary

**Supplementary information**

**Suppl. Fig. 1**: Characterization of circulating extracellular vesicles of healthy and preeclamptic pregnant women


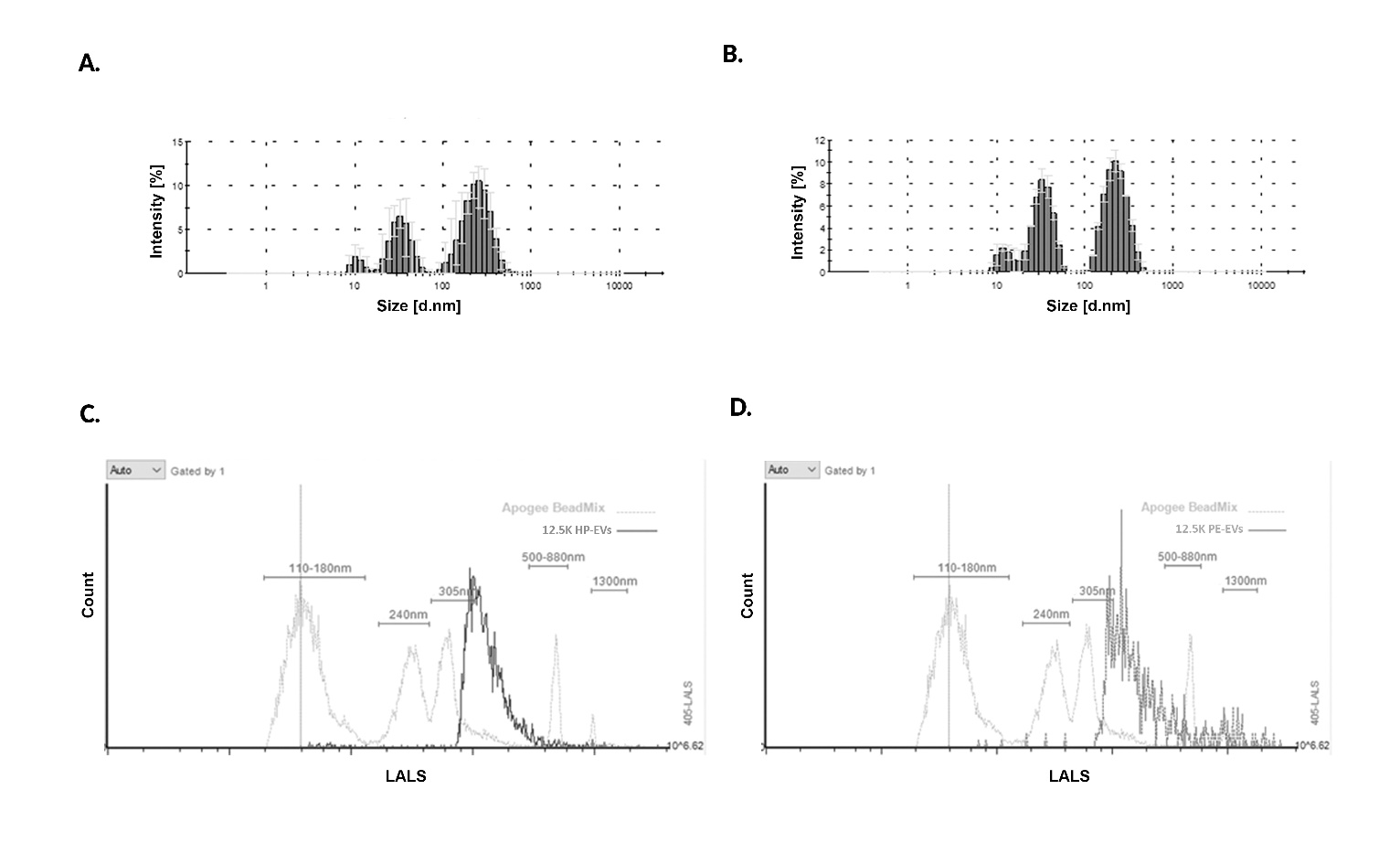


Representative HP-EVs (A. mean size ± SEM: HP-EV=187.4 ± 8.2 nm), PE-EVs (B. mean size ± SEM: PE-EV= 156.6 ± 2.33 nm) size distribution assessed by dynamic light scattering Malvern Zetasizer Nano S (Malvern Instruments Ltd, Malvern, UK). Bars depict at least 3 consecutive measurements (n=6 patient samples/ group). Size distribution of 12.5K pellet (C – HP-EVs; D – PE-EVs) assessed by dedicated high-resolution flow cytometer, representative histograms; LALS – large angle light scatter detector.

**
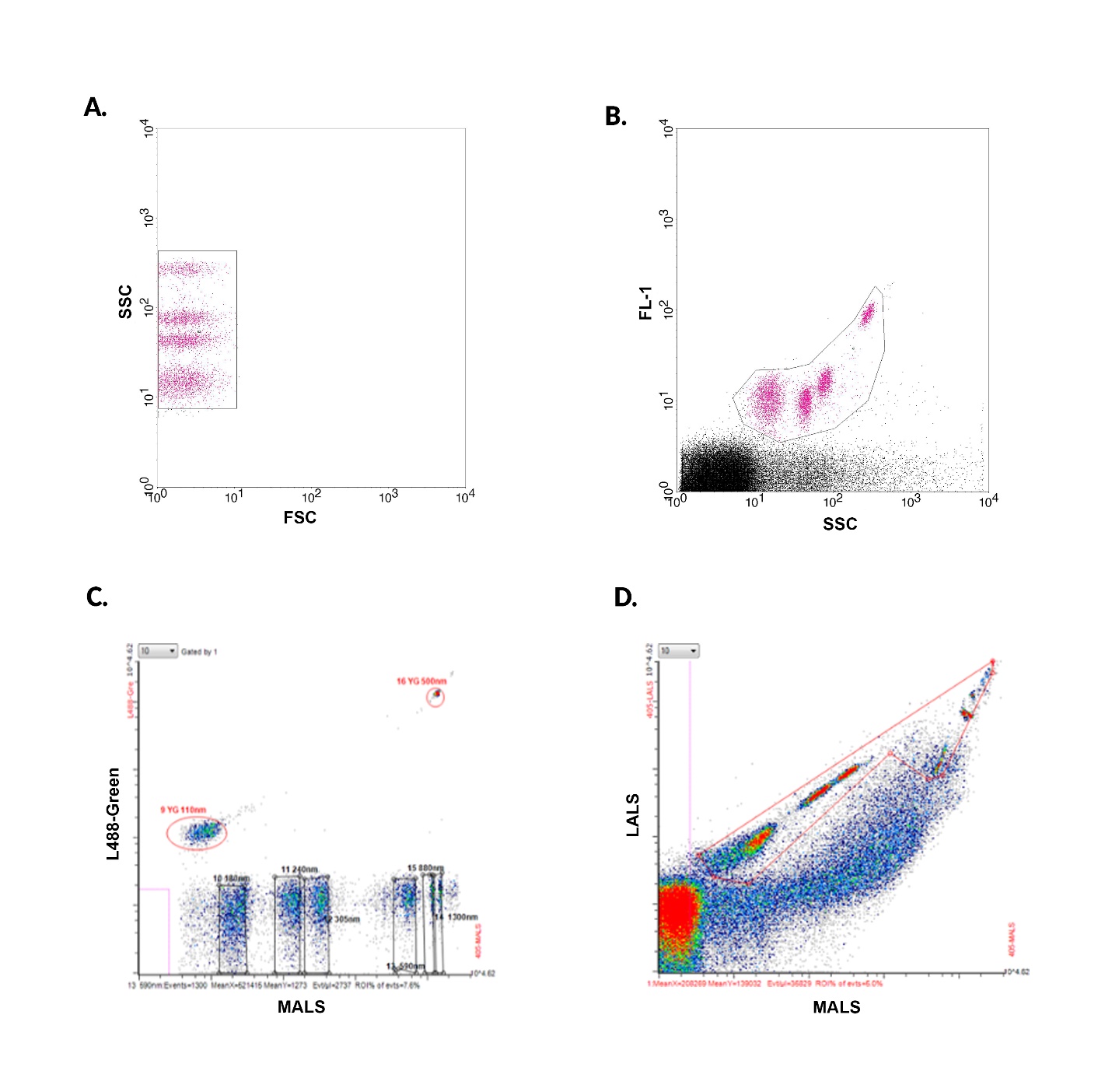
Suppl. Fig. 2:** Gating strategy and triton lysis for conventional and high-resolution flow cytometry

Megamix Beads (Biocytex) for FACSCalibur and Reference Bead Mix (ApogeeMix, Apogee Flow Systems) for Apogee A50 Micro were used both for optimization of cytometer settings and gating strategy. A-B. 160 nm, 200 nm, 240 and 500 nm fluorescent Megamix beads with refractive index (RI) 1.59 were applied to FACSCalibur instrument determine the EV gate through multicolor gating; SSC – side scatter; FSC – forward scatter; FL-1 – fluorescence channel 1(green fluorescence channel (525-530 nm)); . C-D. 180 nm, 240 nm, 300 nm, 590 nm, 880 nm, and 1,300 nm plastic spheres with RI 1.42 or 110 nm and 500 nm green fluorescent beads with RI 1.59 were used to assess the sensitivity and resolution of the A50-Micro flow cytometer and to define EV gate. MALS – middle angle light scatter; LALS – large angle light scatter; L-488-Green – fluorescent channel 1 (green fluorescence channel (525-530 nm)) L488-Green is the “analogue” of the FL-1 channel (Apogee A50Micro). MALS is the “analogue” of the FSC, LALS is the “analogue” of the SSC, respectively.

**
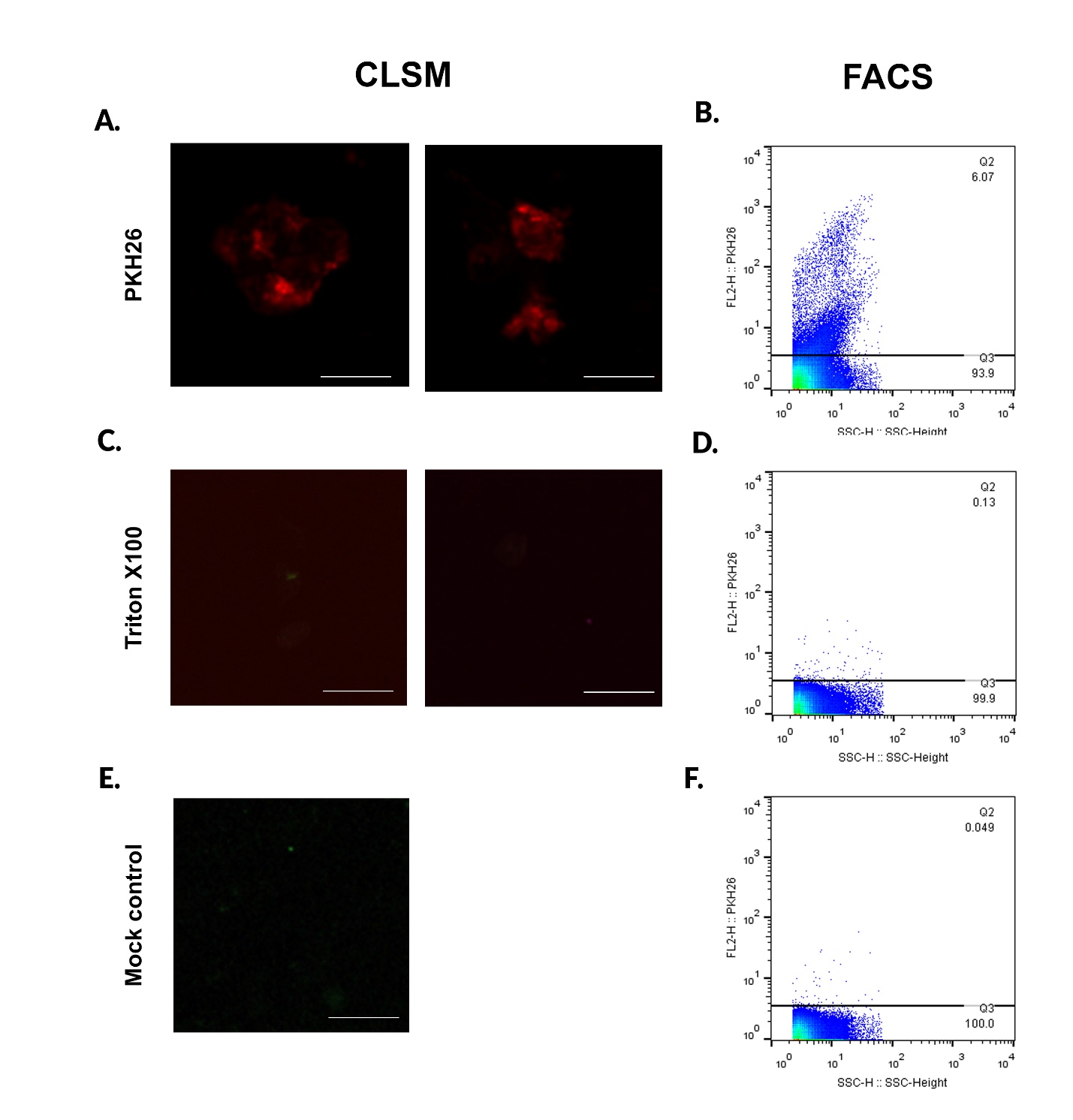
Suppl. Fig. 3:** PKH labelling of EVs measured by CLSM and FACS

PKH26 labelled isolated 12.5K fraction of EVs detected by CLSM (A.) and FC (B.). After applying 0.1% Triton X-100 detergent lysis the vesicle could not be detected either by CLSM (C.) or FC (D.). The mock control containing only the PKH26 dye did not show any fluorescence as observed by CLMS (E.) or FC (F.). Scale bar represents 500 nm.

**
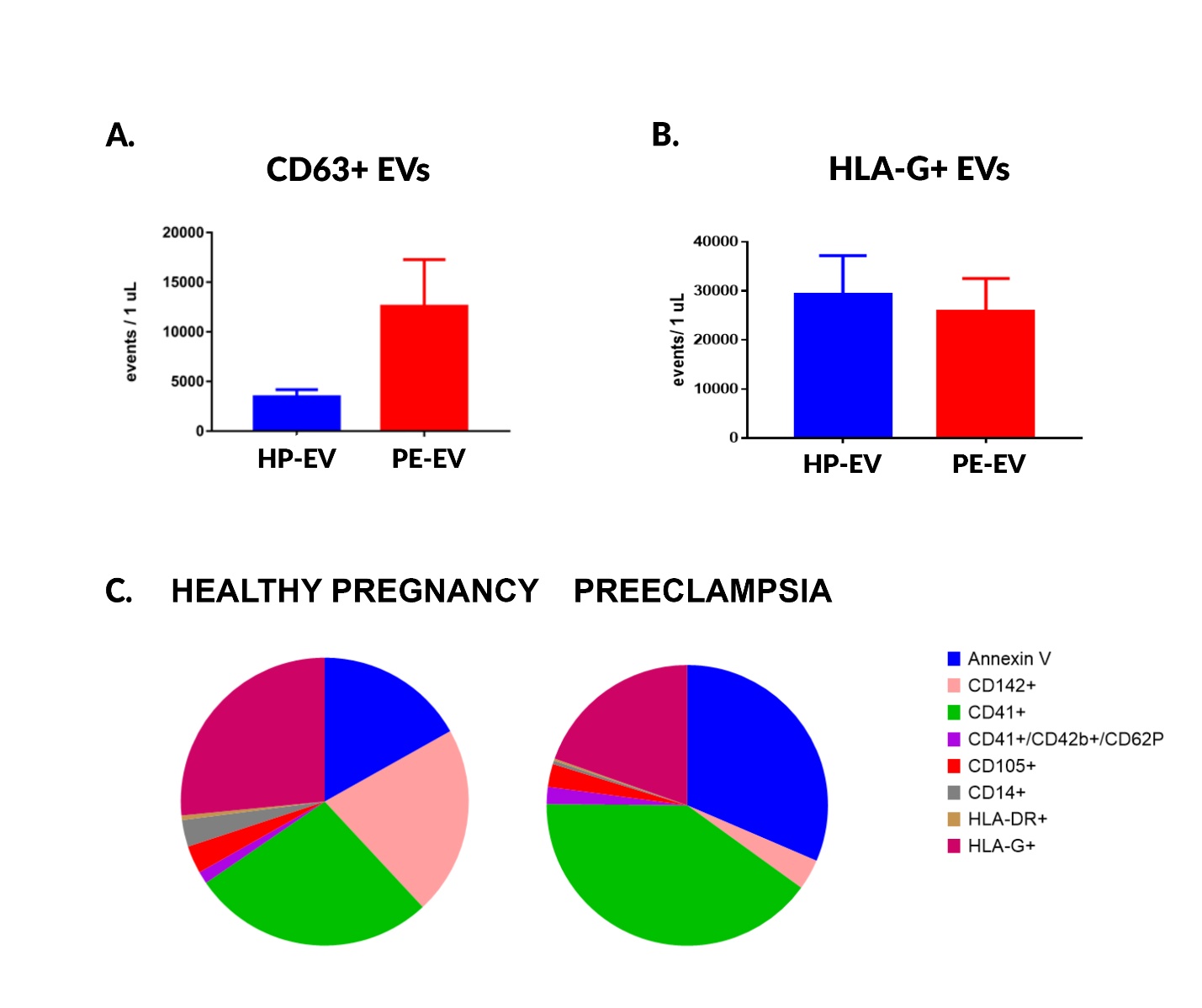
Figure S4:** Immunophenotyping of circulating EVs

A. Number of CD63 positive EV count, as evaluated by FC. CD63 mean values ± SEM: HP-EV = 3461 ± 715 PE-EV = 12778 ± 4698 P>0.05 (n=13). B. Number of HLA-G positive (syncytiotrophoblast-derived) EV count evaluated by FC. HLA-G mean values ± SEM: HP-EVs = 29 348 ± 2 380 EVs / μL PE-EVs = 25 822 ± 2 030 EVs / μL; P>0.05 (n=15) C. Pie chart diagram showing the relative distribution of the most abundant EV subpopulations detected in the EV pool. Annexin V positivity (Phosphatidylserine positive EVs), CD142 positivity (Tissue factor positive EVs), HLA-G positivity (Trophoblast-derived EVs), HLA-DR positivity (MHC II Class positive EVs), CD41 positivity (Platelet-derived EVs), CD41/CD42b/CD62P triple positivity (Activated platelet-derived EVs), CD14 positivity (Monocyte-derived EVs), CD105 positivity (Endothelial cell-derived EVs).

**Suppl.**
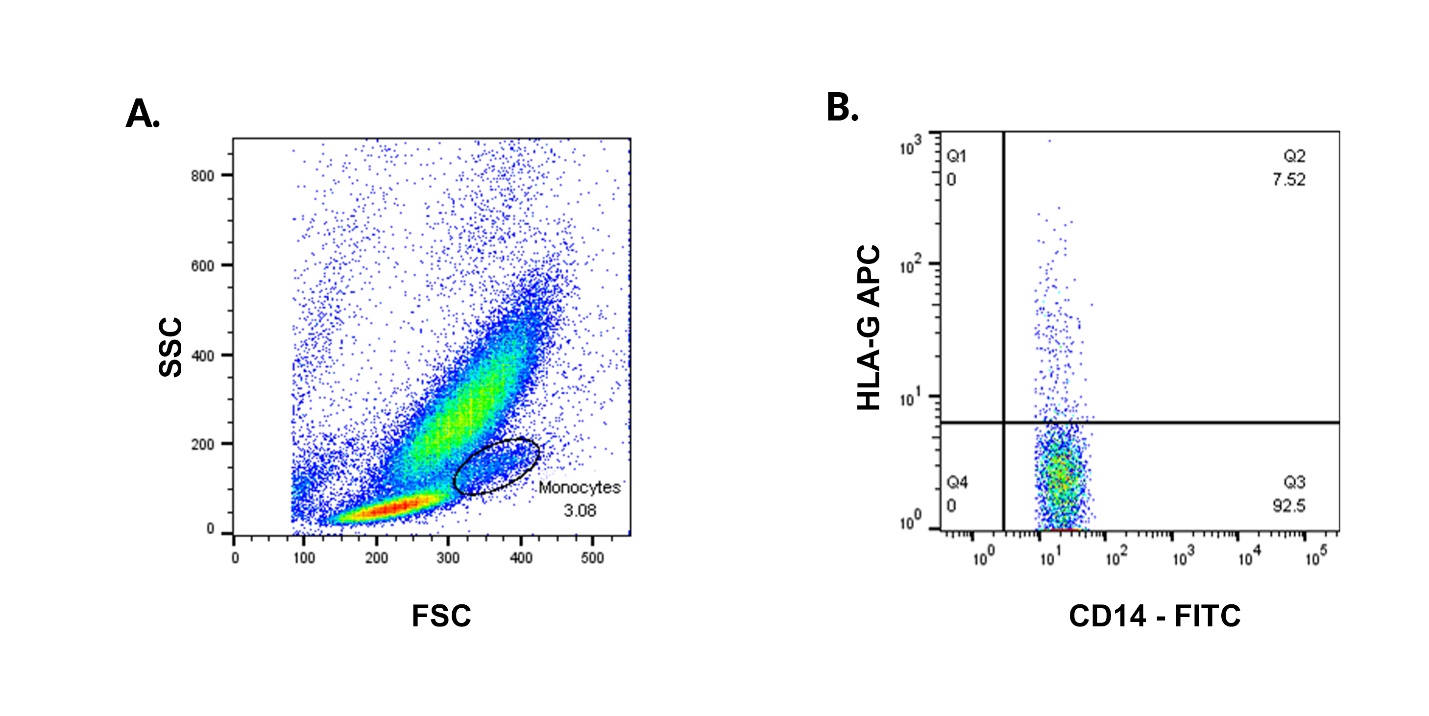
**Fig. 5**: Binding of HLA-G positive circulating trophoblast-derived EV to monocytes

**A.** Representative dot plot of the monocyte gating (confirmed by anti-CD14-FITC staining). **B.** The mean fluorescence intensity of APC conjugated HLA-G is shown within the monocytes gate.


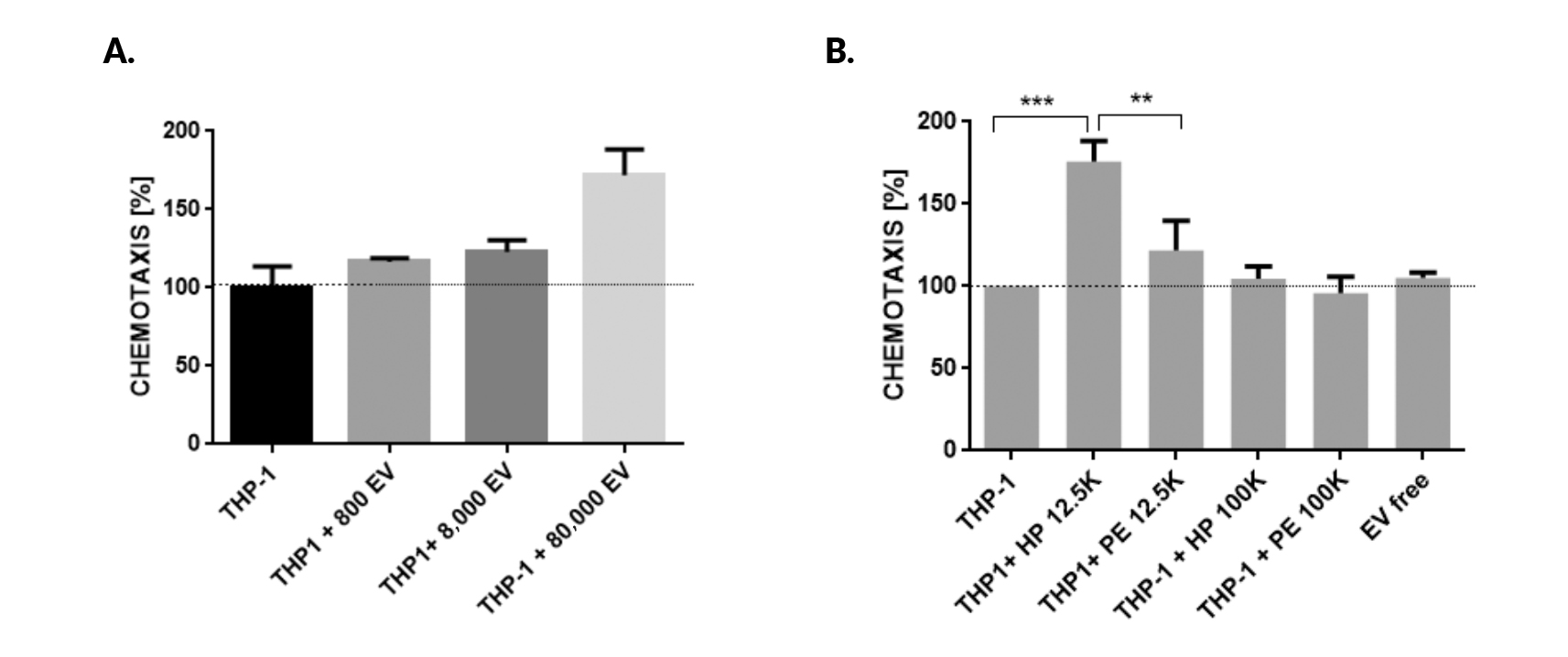
**Suppl. Fig. 6:** Concentration dependency of EV-induced chemotaxis and effects of different EV subpopulations.

A. EVs isolated from pregnant women induce chemotaxis in THP-1 cells in a concentration-dependent manner. THP-1 cells (40,000 cells/ well) were treated with 800, 8,000 and 80,000 12.5K fraction of EVs/ well, respectively. B. Chemotactic effects of the different EV fractions (12.5K, 100K and EV-depleted preparations) on THP-1 cells (n=6 patient samples per group, each patient sample was tested in 9 parallel measurements).

**Suppl. Fig. 7:** IL-6 and TNF relative mRNA expression upon EVs (12.5K and 100K fractions) stimulation

**
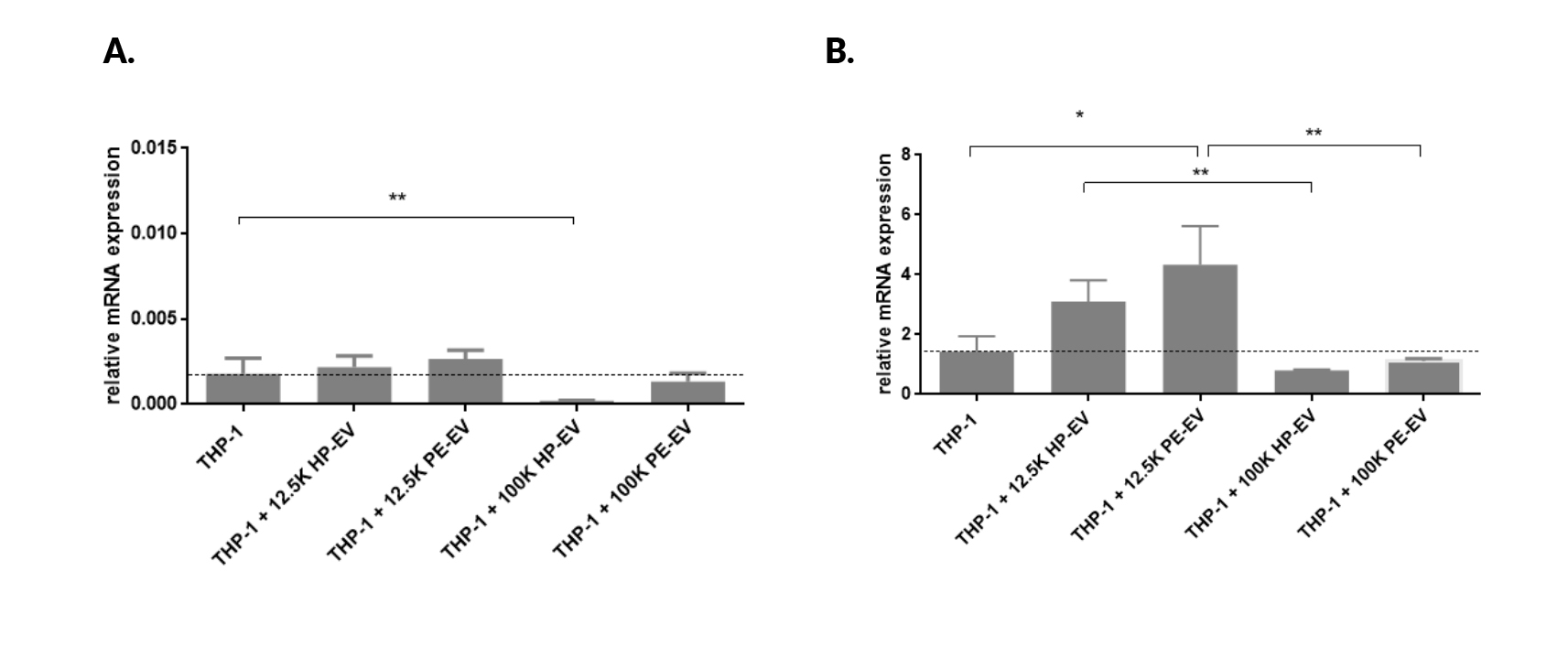
**

A. IL-6 gene expression after 24 hours of incubation can be seen upon different cohorts of EVs (patient samples n=6). B. TNF gene expression after 24 hours of incubation can be seen upon different cohorts of EVs (patient samples n=6).

**Suppl. Table 1:** Migration associated proteins in EVs identified by mass spectrometry

| Protein | Sample | Ligand | Described interaction | Reference |
| --- | --- | --- | --- | --- |
| Migration associated proteins | | | | |
| RAP2A | PE-MV only | p-Akt | Overexpression enhances migration and invasion in cancer cells | ^1^ |
|  |  | RAPL | Activates random T cell migration | ^2^ |
| BCAM | PE-MV only | Lamin α5 | Promotes tumor cell migration | ^3^ |
| BSG | PE-MV only | Galectin3, E-selectin | Migration of inflammatory leukocytes, induction of matrix metalloproteinases | ^4^ |
| THBS4 | PE-MV only | Integrin αMβ2 and β3 | Increasing the migration of macrophages in dose dependent manner, proangiogenic activity | ^5,6^ |
| ITGA6 | HP-MV only | Laminin | Increased migration of mesenchymal stem cells | ^7^ |
|  |  | Laminin | Promotes migration in macrophages | ^8^ |
| CDC42 | HP-MV only | Actin | Regulates the direction of migration and the polymerization of actin to form peripheral lamellipodial and filopodial protrusions, controls directionality | ^9^ |
| RAC1 | HP-MV only | Filamin A | Directly regulates migration in monocytes | ^10^ |
| MYH9 | BOTH HP AND PE MV | Dlc1 | Phosphorylated Myh9 plays a key role in cell migration | ^11^ |
|  |  | Rac1 | Acts as a critical modulator of cell migration in integrin-dependent manner | ^12^ |
| ITGB3 | BOTH HP AND PE MV | ICAM-1 | Engage monocytes in stronger migration | ^13^ |
| MMRN1 | BOTH HP AND PE MV | Collagen type I | Supports human platelet adhesion | ^14^ |
| CD36 | BOTH HP AND PE MV | PS | EV inhibit endothelial cell migration via CD36 and phosphatidylserine (PS) interaction | ^15^ |
| MMP9 | BOTH HP AND PE MV |  | The migration of mature monocyte-derived dendritic cells is controlled by the balance of MMP9 and TIMPs | ^16^ |
| CD99 | BOTH HP AND PE MV | CD99 | Homophilic interaction facilitates transmigration of monocytes at endothelial cell junctions. | ^17^ |
| RHOA | BOTH HP AND PE MV |  | Promotes actin:myosin contraction in the cell body and at the rear | ^9^ |
| Adhesion associated proteins | | | | |
| ICAM3 | PE-MV only | LFA1 | Increased adhesion, modulating macrophage/ monocyte recruitment. | ^18^ |
| RAP2A | PE-MV only | LFA1, integrin α4 | Enhanced B cell adhesion | ^19^ |
| TGFBI | PE-MV only | Fibronectin | Overexpression of TGFBI reduces adhesion and migration in HSPCs | ^20^ |
| THBS4 | PE-MV only | Integrin αMβ2 and β3 | Increasing the adhesion of macrophages in dose-dependent manner | ^5,6^ |
| ITGA6 | HP-MV only | Laminin | Decreased adhesion to laminins of mesenchymal stem cells | ^7^ |
| RAC1 | HP-MV only | MYH9 | Rac1 activity promotes capture and assembly of Myh9 mini-filaments in maturing focal adhesions | ^12^ |
| CD11b | BOTH HP AND PE MV | Fibrinogen,  I-CAM1 | Blocking of CD11b enhances the adhesion of monocytes derived dendritic cells and macrophages | ^21^ |
| THBS1 | BOTH HP AND PE MV | VCAM | Up-regulates monocyte adhesion to endothelium | ^22^ |
| TLN1 | BOTH HP AND PE MV | Integrin α4β1 | Participates in the later stage of leukocyte adhesion cascade, contributing to adhesion strengthening. | ^23^ |
| Phagocytosis associated proteins | | | | |
| RAC1 | HP-MV only |  | Rac1 and Rac2 differ in precise localization and time of activation at phagocytic cups | ^24^ |
| RAC2 | PE-MV only |  |  |  |
| TGFBI | PE-MV only |  | After ingestion of apoptotic cells, monocyte-derived macrophages produce TGF beta-induced (TGFBI) and activate the collagen production in fibroblasts | ^25^ |
| THBS1 | BOTH HP AND PE MV |  | Mediates macrophage phagocytosis of apoptotic cells via CD36 | ^26^ |

1. Wu, J.-X., Zhang, D.-G., Zheng, J.-N. & Pei, D.-S. *Rap2a is a novel target gene of p53 and regulates cancer cell migration and invasion.* *Cellular signalling* **27,** (2015).

2. Miertzschke, M. *et al.* Characterization of interactions of adapter protein RAPL/Nore1B with RAP GTPases and their role in T cell migration. *J. Biol. Chem.* **282,** 30629–30642 (2007).

3. Kikkawa, Y. *et al.* The lutheran/basal cell adhesion molecule promotes tumor cell migration by modulating integrin-mediated cell attachment to laminin-511 protein. *J. Biol. Chem.* **288,** 30990–31001 (2013).

4. Muramatsu, T. Basigin (CD147), a multifunctional transmembrane glycoprotein with various binding partners. *Journal of Biochemistry* **159,** 481–490 (2016).

5. Frolova, E. G. *et al.* Thrombospondin-4 regulates vascular inflammation and atherogenesis. *Circ. Res.* **107,** 1313–1325 (2010).

6. Muppala, S. *et al.* Proangiogenic properties of thrombospondin-4. *Arterioscler. Thromb. Vasc. Biol.* **35,** 1975–1986 (2015).

7. Yang, Z. *et al.* CD49f Acts as an Inflammation Sensor to Regulate Differentiation, Adhesion, and Migration of Human Mesenchymal Stem Cells. *Stem Cells* **33,** 2798–2810 (2015).

8. Shaw, L. M. & Mercurio, A. M. Regulation of alpha 6 beta 1 integrin-mediated migration in macrophages. *Agents Actions. Suppl.* **47,** 101–106 (1995).

9. Raftopoulou, M. & Hall, A. Cell migration: Rho GTPases lead the way. *Developmental Biology* **265,** 23–32 (2004).

10. Leung, R. *et al.* Filamin A regulates monocyte migration through Rho small GTPases during osteoclastogenesis. *J. Bone Miner. Res.* **25,** 1077–1091 (2010).

11. Erfani, N. *et al.* Dlc1 interaction with non-muscle myosin heavy chain II-A (Myh9) and Rac1 activation. *Biol. Open* **109,** 808–817 (2016).

12. Pasapera, A. M. *et al.* Rac1-dependent phosphorylation and focal adhesion recruitment of myosin IIA regulates migration and mechanosensing. *Curr. Biol.* **25,** 175–186 (2015).

13. Weerasinghe, D. *et al.* A role for the αvβ3 integrin in the transmigration of monocytes. *J. Cell Biol.* **142,** 595–607 (1998).

14. Reheman, A., Tasneem, S., Ni, H. & Hayward, C. P. M. Mice with deleted multimerin 1 and ??-synuclein genes have impaired platelet adhesion and impaired thrombus formation that is corrected by multimerin 1. *Thromb. Res.* **125,** (2010).

15. Ramakrishnan, D. P., Hajj-Ali, R. A., Chen, Y. & Silverstein, R. L. Extracellular vesicles activate a CD36-dependent signaling pathway to inhibit microvascular endothelial cell migration and tube formation. *Arterioscler. Thromb. Vasc. Biol.* **36,** 534–544 (2016).

16. Osman, M., Tortorella, M., Londei, M. & Quaratino, S. Expression of matrix metalloproteinases and tissue inhibitors of metalloproteinases define the migratory characteristics of human monocyte-derived dendritic cells. *Immunology* **105,** 73–82 (2002).

17. Gerhardt, T. & Ley, K. Monocyte trafficking across the vessel wall. *Cardiovascular Research* **107,** 321–330 (2015).

18. Torr, E. E. *et al.* Apoptotic cell-derived ICAM-3 promotes both macrophage chemoattraction to and tethering of apoptotic cells. *Cell Death Differ.* **19,** 671–9 (2012).

19. McLeod, S. J., Shum, A. J., Lee, R. L., Takei, F. & Gold, M. R. The Rap GTPases Regulate Integrin-mediated Adhesion, Cell Spreading, Actin Polymerization, and Pyk2 Tyrosine Phosphorylation in B Lymphocytes. *J. Biol. Chem.* **279,** 12009–12019 (2004).

20. Klamer, S. E. *et al.* BIGH3 modulates adhesion and migration of hematopoietic stem and progenitor cells. *Cell Adh. Migr.* **7,** 434–49 (2013).

21. Sándor, N. *et al.* CD11c/CD18 Dominates Adhesion of Human Monocytes, Macrophages and Dendritic Cells over CD11b/CD18. *PLoS One* **11,** e0163120 (2016).

22. Narizhneva, N. V *et al.* Thrombospondin-1 up-regulates expression of cell adhesion molecules and promotes monocyte binding to endothelium. *FASEB J.* **19,** 1158–1160 (2005).

23. Hyduk, S. J. *et al.* Talin-1 and kindlin-3 regulate alpha4beta1 integrin-mediated adhesion stabilization, but not G protein-coupled receptor-induced affinity upregulation. *J Immunol* **187,** 4360–4368 (2011).

24. Mao, Y. & Finnemann, S. C. Regulation of phagocytosis by Rho GTPases. *Small GTPases* **6,** 1–11 (2015).

25. Nacu, N. *et al.* Macrophages produce TGFβ1 (BIGH3) following ingestion of apoptotic cells and regulate MMP14 levels and collagen turnover in fibroblasts. *J. Immunol.* **180,** 5036–44 (2008).

26. Gutierrez, L. S., Lopez-Dee, Z. & Pidcock, K. Thrombospondin-1: Multiple paths to inflammation. *Mediators of Inflammation* **2011,** (2011).

27. Bodnar, L. M., Ness, R. B., Markovic, N. & Roberts, J. M. The risk of preeclampsia rises with increasing prepregnancy body mass index. *Ann. Epidemiol.* **15,** 475–482 (2005).

28. Sohlberg, S. et al. Maternal body mass index, height, and risks of preeclampsia. *Am. J. Hypertens.* **25,** 120–5 (2012).

**Suppl. Table 2 -**. Patient clinical data

|  | Healthy 3^rd^ trimester pregnants (n=20) | Preeclamptic 3^rd^ trimester pregnants (n=25) |
| --- | --- | --- |
| Maternal age (mean ± SD) | 33.8 ± 4.7 years | 31.7 ± 4.6 years |
| Gestational age at sampling (mean ± SD) | 33.8 ± 3.5 weeks | 32.0 ± 4.3 weeks |
| Gestational age at birth (mean ± SD) | 38.9 ± 1.2 weeks | 32.5 ± 4.3 weeks |
| Birth weight (mean ± SD) | 3560 ± 416 g* | 1688 ± 922 g* |
| Early onset PE (%) | not applicable | 48% |
| HELLP syndrome (%) | 0% | 19% |
| BMI (mean ± SD) | 22.3 ± 3.1 | 26.3 ± 7.6 |
| Systolic/diastolic blood pressure (mean ± SD) | 115.8 ± 5.3 / 71.4 ± 3.1 mm Hg ** | 155.0 ± 18.4 / 94.1 ± 13.9 mm Hg ** |
| Urine protein (mean ± SD) | not detectable | 4063± 2329 mg/24 h |

For the in vitro experiments, at least 6 blood plasma samples were chosen both from preeclamptic and healthy groups. There was no significant difference between the healthy and preeclamptic pregnant women group regarding maternal age and gestational age at sampling. Nineteen % of women with diagnosed preeclampsia developed HELLP syndrome and 5 % of them (1 patient) progressed into eclampsia later during pregnancy. In accordance with other studies, higher BMI index was detected in the preeclamptic group ^27,28^. The gestational age at labour was significantly lower in preeclamptic group. Comparison of neonatal birth weight and gestational age at birth showed differences between healthy pregnants and preeclamptic patients. *Significantly lower birth weight was detectable in case of newborns of preeclamptic pregnants, unpaired *t*-test, p<0.0001; **Significantly elevated blood pressure was observable in the preeclamptic group, Mann Whitney U test p<0.001

**Suppl. Fig. 8** Proteomic analysis of circulating extracellular vesicles (MS) n=7 per group.

**
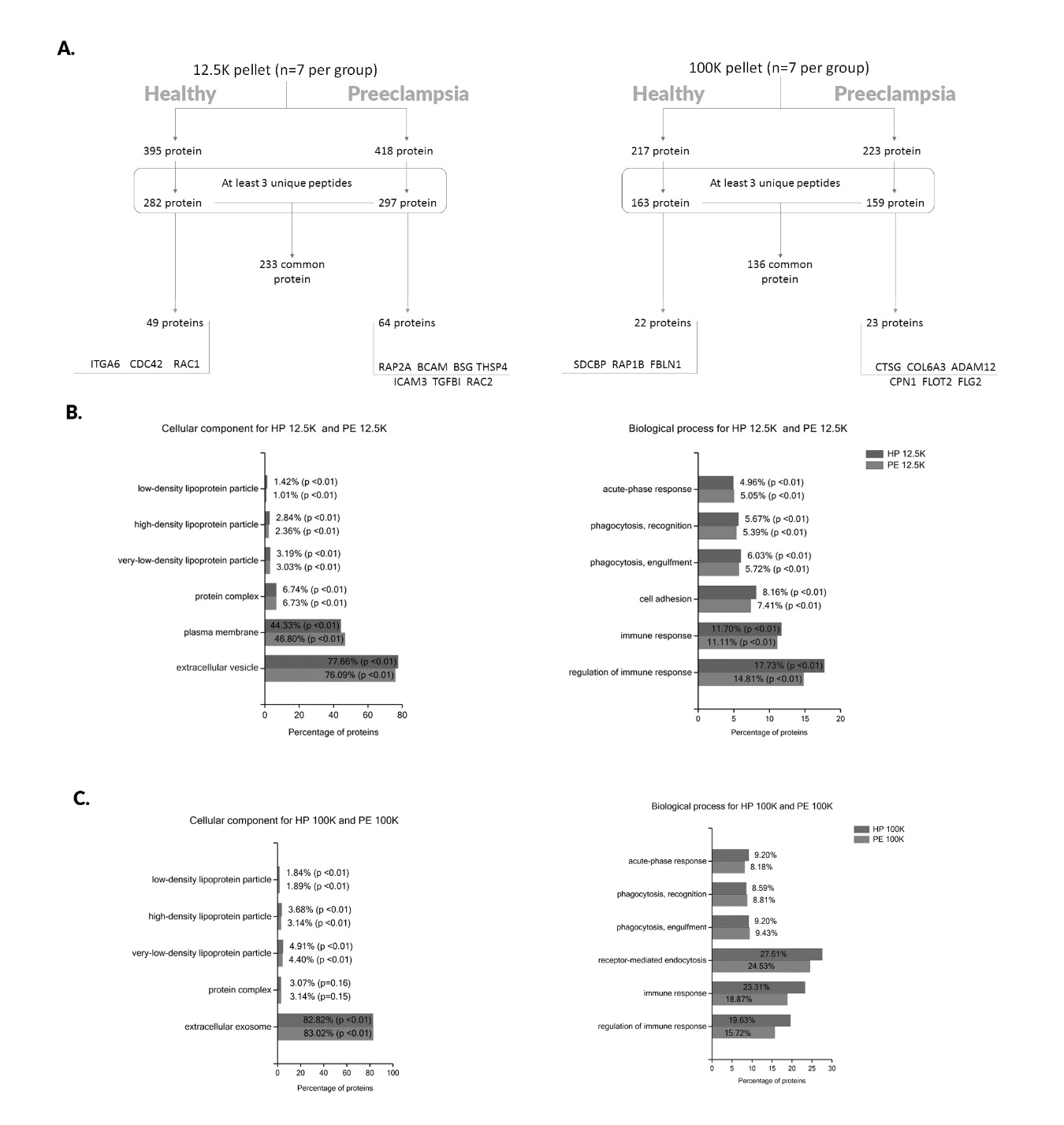
**

A. Following the algorithm for the analysis of proteomic data, key proteins playing significant role in phagocytosis, cell migration and adhesiveness were observed exclusively in healthy or preeclamptic 12.5K fraction of EVs. B. and C. Term enrichment analysis by cellular component and biological processes reveals no difference between PE-EVs and HP-EVs. There was not any significant difference between PE-EV and HP-EV fractions regarding the identified possible contaminating lipoprotein (the co-purified LDL, HDL particles) and co-purified protein complexes.

**Suppl. Fig. 9:** EV isolation schematic diagram

**
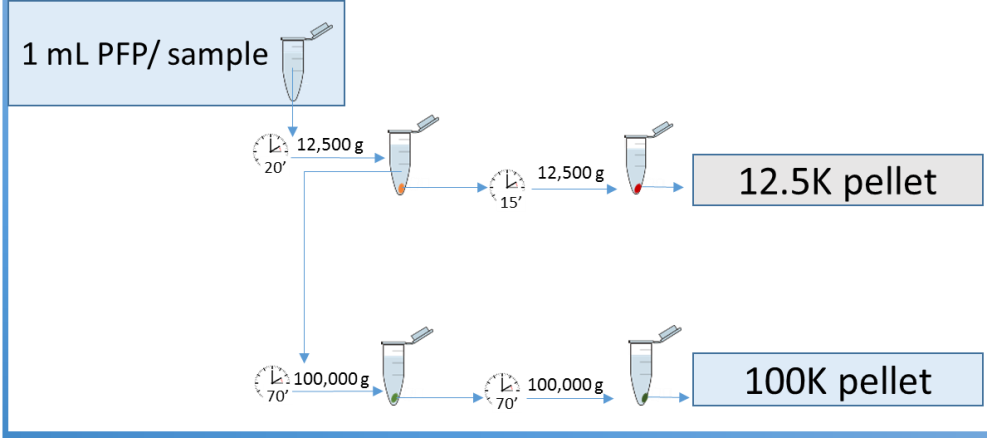
**

EV isolation steps during differential ultracentrifugation: 1 mL of PFP sample is centrifuged at 12,500 *g* for 20 minutes and the obtained pellet is subsequently washed by filtered PBS (through 0.22 μm). Correspondingly the supernatant is further centrifuged at 100,000 *g* for 70 minutes and the pellet subsequently is also washed by filtered PBS. The obtained 12.5K EV pellets are enriched in “microvesicles”, 100K EV pellets are enriched in “exosome” fractions.

**Suppl. Tab. 3** List of antibodies and dyes used for immunofluorescence studies

| **Antibody/Dye name** | **Clone*** | **Manufacturer** | **Catalog number** | **Applied Methods** |
| --- | --- | --- | --- | --- |
| PE anti-human HLA-G | 87G | Biolegend | 335906 | FACS – EV |
| APC anti-human HLA-G | 87G | eBioscience | 17995742 | FACS EV binding |
| FITC Annexin V |  | Biolegend | 640906 | FACS-EV,CLSM- EV |
| PE Annexin V |  | SONY | 3804540 | FACS-EV, CLSM- EV |
| APC Annexin V |  | SONY | 3804600 | FACS-EV |
| PE anti-human CD63 | MEM 259 | Sigma | SAB4700218 | FACS-EV |
| PerCP anti-human CD9 | M-L13 | BD | 561329 | FACS-EV |
| FITC anti-human CD41a | HIP8 | BD | 555466 | FACS – EV |
| APC anti-human CD62P | AK-4 | BD | 555523 | FACS – EV |
| PE anti-human CD142 | NY2 | SONY | 2426015 | FACS – EV |
| PE anti-human CD105 | SN6h | SONY | 4602520 | FACS-EV |
| PerCP anti-human HLA-DR | L243 | BD | 339216 | FACS – EV |
| PKH26 |  | Sigma | P9691 | FACS-EV, Phagocytosis and Binding Assay, CLSM- EV |
| AF488 anti-human CD47 | 1/1A4 | AbD Serotec | MCA2514A488T | FACS – EV |
| FITC anti-human TNFa | Mab11 | eBioscience | 11734941 | FACS – THP-1 intracellular staining |
| FITC anti-human CD14 | 61D3 | eBioscience | 11014942 | FACS – PBMC |

* In case of antibodies; FACS – EV – flow cytometry of extracellular vesicles; CLSM-EV – confocal laser scanning microscopy of extracellular vesicles; FACS-PBMC – monocyte immunophenotyping

**Suppl. Fig. 10** THP-1 cell viability – FC, Light microscopy

**
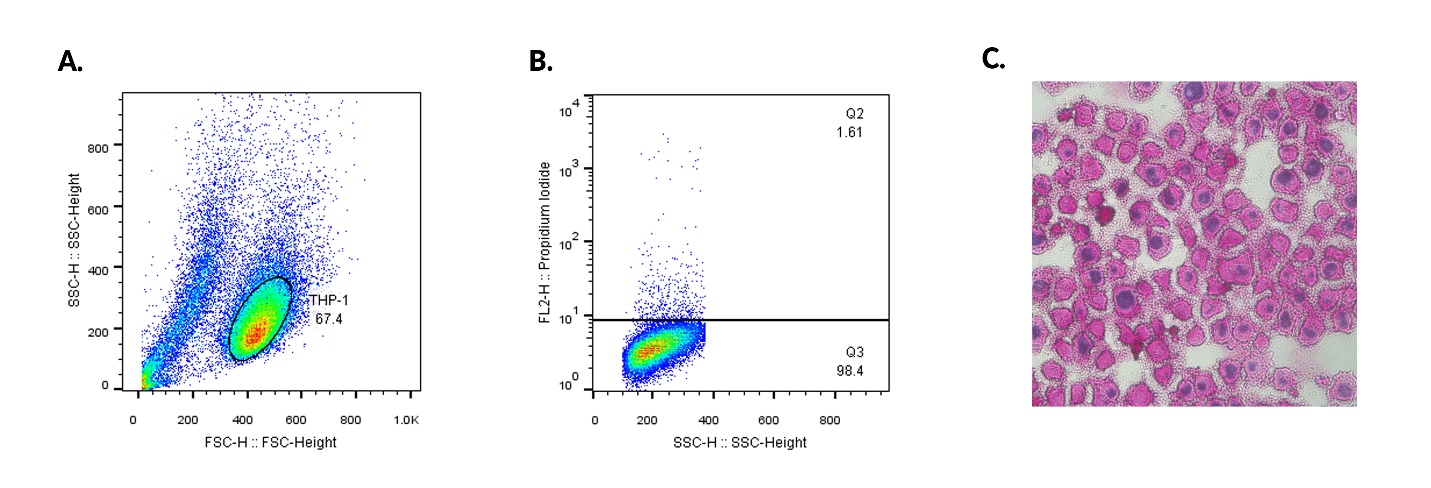
**

Cultures of THP-1 were maintained in RPMI-1640 (Sigma Ltd. St. Luis, MP, USA) containing 10% FCS (Gibco, New York, NY, USA), L-glutamine (2mM), 100 μg/mL penicillin/streptomycin (Gibco/Invitrogen Corporation, New York, NY, USA), 1% HEPES, 2-mercaptoethanol (0.010 mM). Cells were maintained under standard culture conditions at 37C in humidified 5% CO_2_ atmosphere with medium renewal every 2 days and regularly screened for bacterial or yeast infection. THP-1 cells prior to EV treatment were maintained for 24 h on serum free medium. The cell viability under serum free medium conditions was not altered: A. Representative FSC/SSC dot plot shows the morphology (size and granulation) of cultured THP-1 cells, and the definition of “living cell” gate. B. SSC/FL2 dot plot represents the PI uptake of THP-1 cells, inside the “living cell” gate. C. Representative May-Grünwald Giemsa staining of cultured THP-1 cells.

**
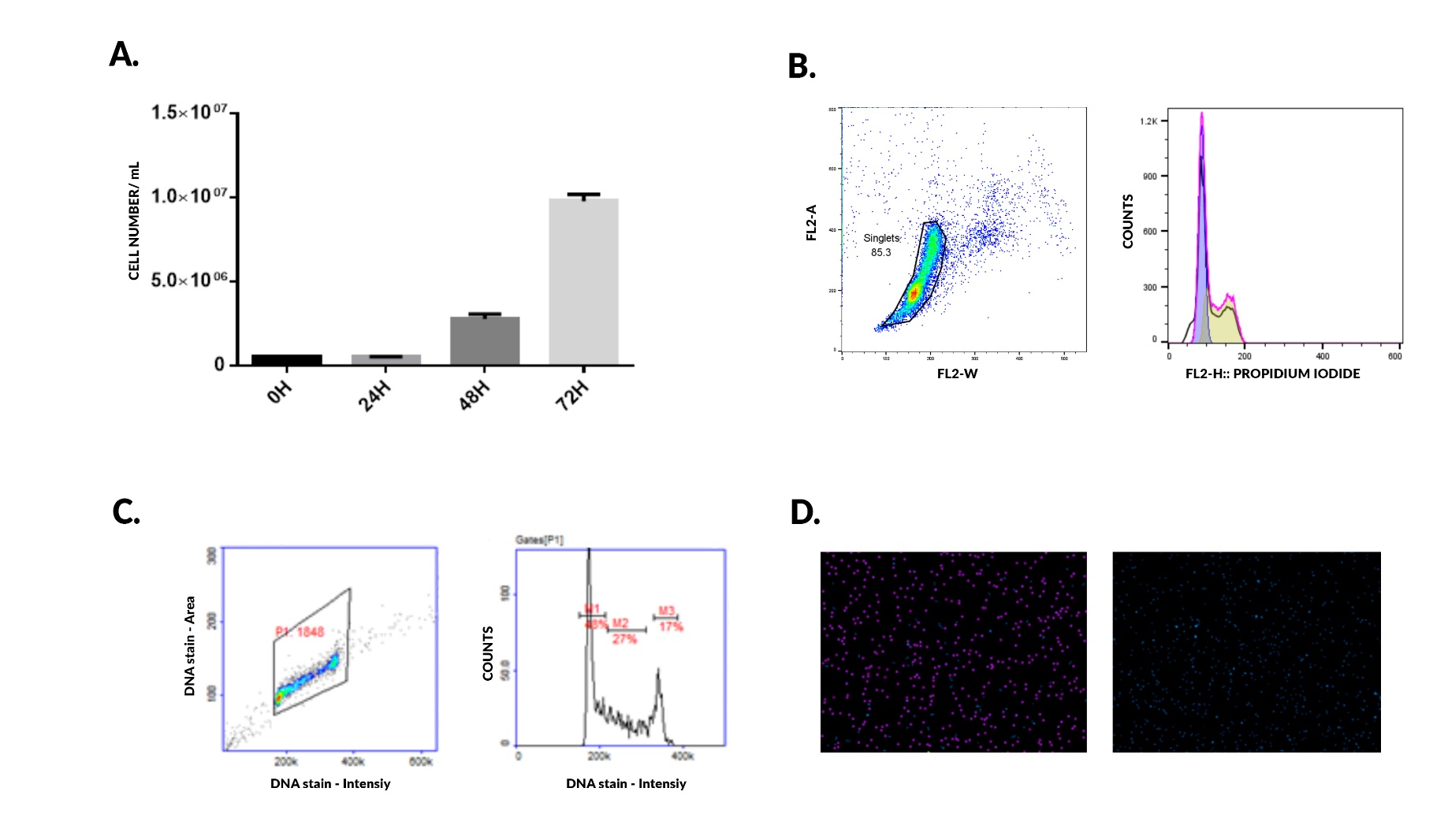
Suppl. Fig. 11:** Population doubling time and cell cycle analysis of resting THP-1 cells

A. Population doubling time measurement of resting THP-1 cells. The cell numbers were counted after 24, 48 and 72 hours of seeding by flow cytometry (PKH26 reference bead, Sigma, St Louis, MO, USA). During the experiments THP-1 cells were cultured in standard culture conditions (n=6). B. Representative cell cycle analysis measured with propidium iodide stain by a flow cytometric approach. C. Representative dot plot and histogram of cell cycle analysis measured by DAPI stain with automated cell analyzer NucleoCounter (NC-250, Chemometec, Denmark) (Validation of the flow cytometric measurements). D. Representative images show fluorescence intensity for precise definition of the events to count for cell cycle analysis by NucleoCounter.
